# Do you say *uh* or *uhm*? A cross-linguistic approach to filler particle use in heritage and majority speakers across three languages

**DOI:** 10.3389/fpsyg.2024.1305862

**Published:** 2024-03-11

**Authors:** Marlene Böttcher, Margaret Zellers

**Affiliations:** Department of General Linguistics and Phonetics, Institute for Scandinavian Studies, Frisian Studies and General Linguistics, Kiel University, Kiel, Germany

**Keywords:** filler particles, heritage speaker, bilingualism, speech planning, speech corpora, English, Russian, German

## Abstract

Filler particles like *uhm* in English or *ähm* in German show subtle language-specific differences and their variation in form is related to socio-linguistic variables like gender. The use of fillers in a second language has been shown to differ from monolinguals' filler particle use in both frequency and form in different language contexts. This study investigates the language-specific use of filler particles by bilingual heritage speakers in both their languages, looking at the dominant majority language in the society and their minority heritage language spoken at home. This is done based on heritage Russian and German data and majority German and English data from the RUEG corpus. Language-specific fillers were extracted from the corpus and analyzed for their occurrence and segmental form. The frequency analysis suggests an influence of bilingualism, age group, and formality of the situation on the filler frequency across all languages. The number of filler particles is higher in formal, older, and bilingual speech. The form analysis reveals an effect of language and gender on the type of filler particle. The vocalic-nasal filler particles (e.g., *uhm*) are more frequently found in German and English and in female speech of these languages. Heritage speakers of Russian in contact with German and English show higher use of vocalic-nasal forms also in their Russian while producing similar gender related patterns to monolingual speakers in both their languages. The higher frequency of filler particles in formal situations, older speakers and in bilingual speech, is discussed related to cognitive load which is assumed to be higher in these contexts while speech style which differs between situations and social groups is also considered as explanation. The higher use of vocalic-nasal filler particles in German and English suggests language specific filler particle preferences also related to the socio-linguistic variable gender in these languages. The results from heritage speakers suggest and influence on filler particle form in their heritage language, while also revealing socio-linguistic usage patterns related to gender which are produced by heritage speakers similarly to monolinguals in their respective language.

## 1 Introduction

So-called filler particles, like the constituents *uh* and *uhm* (and their phonetic variants) in English, are one type of disfluency, along with repetitions, repairs and silent pauses. They are aspects of spontaneous discourse and have been reported to comprise about ten percent of words in natural conversations (Shriberg, 2001).

The majority of prior work has focused on filler particles either as symptoms and thus on their status as hesitations, or on fillers as signals in discourse at minor and major discourse boundaries, indicating e.g. discourse structure or turn management. Terms referring to fillers as symptoms, like *hesitation, disfluency* or *error*, reflect their interpretation as being related to speech planning and their negative reputation in opposition to fluent speech free of such errors (Levelt, 1983; Shriberg, 2001; Corley and Stewart, 2008; Gilquin, 2008). The use of filler particles has in this work been found to be interpreted by listeners as reflecting higher production difficulty, dishonesty and discomfort (FoxTree, 2002). A moderate use of filler particles, however, has also been connected to higher politeness and charisma (Fischer et al., 2017). Other researchers have used more neutral terms like *filled pause* (Maclay and Osgood, 1959; Rochester, 1973; Berthold and Jameson, 1999; Crible et al., 2017) and *discourse particle* (Fischer, 2000; Pistor, 2016). The choice of terminology reflects researchers' approaches, stressing their similarity with silent pauses in the speech signal in the case of the term “filled pause”, and the fact that phenomena like *uh* and *uhm* are used as signals in discourse management in the case of the term “discourse particle” (Maclay and Osgood, 1959; Rochester, 1973; Fischer, 2000; Clark and FoxTree, 2002; Kjellmer, 2003). We use the term *filler particle* in line with Belz (2021) and Belz (2023) to refer to “segmentally structured, semantically empty and syntactically unconstrained” (Belz, 2023, 6) particles which are frequently produced in naturally occurring speech. In general, filler particles (FPs) have been shown to be multi-functional and their use is connected to speech planning and language processing as well as discourse organization (Clark and FoxTree, 2002). Research on speech planning in bilinguals has provided insight in the area of processing and cognition. In many everyday situations, the availability of two languages in bilinguals requires higher cognitive load in monitoring their speech compared to monolinguals (Kroll and Gollan, 2014). In monolingual mode, bilinguals need to inhibit one language choosing the appropriate linguistic structures in communication [see Bialystok (2017) for an overview of studies and theories on bilingual cognition]. Higher cognitive load can be related to different hesitation phenomena including FPs (Kroll and Gollan, 2014; de Jong, 2018; Betz et al., 2023). At the same time FPs show language-specific forms [e.g. in vowel quality, (Candea et al., 2005) and in segmental form/preference (Clark and FoxTree, 2002)]. The use of FPs has been addressed in more recent studies on bilingualism (Gilquin, 2008; Rose and Watanabe, 2019; de Boer and Heeren, 2020; Lo, 2020; Muhlack, 2023) and is yet to be considered broadly in heritage language research (Polinsky, 2018). This gap will be addressed in this study.

### 1.1 Filler particles

In their work Clark and FoxTree (2002) suggest classifying FPs like *uh* and *uhm* as words “with conventional phonological shapes and meanings […] governed by the rules of syntax and prosody” (Clark and FoxTree, 2002, p.75). While there are cross-linguistic tendencies in the segmental form of FPs, which often consist of a central or centralized vowel quality followed by an optional nasal (Shriberg, 2001; Clark and FoxTree, 2002; de Leeuw, 2007; Lickley, 2015), the quality of this central vowel is language-specific (Candea et al., 2005; Stepanova, 2007; Vasilescu et al., 2007; de Boer and Heeren, 2020; Belz, 2021). In a corpus study Candea et al. (2005) investigated the vowel quality within FPs in eight languages (Arabic, Mandarin Chinese, French, German, Italian, European Portuguese, American English and Latin American Spanish) and found language-specific normalized formant values which can be related to the respective vowel system within a language. Vowels in FPs are therefore not simply the result of a so called “articulatory rest position” (Candea et al., 2005, p.51) but anchored within the language's phonology.

On the segmental level, a dichotomy between a vocalic and a vocalic-nasal form for FPs has been established for several languages [e.g. English (Shriberg, 2001; Clark and FoxTree, 2002; Kjellmer, 2003), German (Fischer et al., 2017; Niebuhr and Fischer, 2019), Danish (Navarretta, 2015); an overview of more language-specific forms can be found in Clark and FoxTree (2002)]. The preference for one of the FP forms seems to be language-specific. While German and English show a tendency for higher VN to V ratios (de Leeuw, 2007; Wieling et al., 2016) there seems to be a V preference e.g. in French (Torreira et al., 2010) and Dutch (de Leeuw, 2007). The following examples are taken from the corpus data investigated in this study and illustrate FP use in the beginning of a narration in (1) English, (1a) Russian and (1b) German. The FPs in the examples are presented in capital letters.

(1) a. Hi I‘m calling about the UHM UH accident.
  b. Да Э  здравствуйте я Э  звоню   насчёт аварии yes FP hello        I FP call-1SG about   accident “Yes UH hello, I am UH calling about an accident.”
  c. ÄHM ja nen schönen,        guten        Tag. FP     yes DET beautiful-DAT.SG good-DAT.SG day “UHM yes, good day.”

While the English example illustrates both a VN form (UHM) and a V form (UH), the Russian example shows two instances of a V variant (Э) and the German example only one VN variant (ÄHM). The transcriptions are language specific and are oriented in thee language specific pronunciation of FP vowels.

Additionally, the two forms have been related to socio-linguistic variation and the variables gender and age. In a corpus study, Acton (2011) investigated the use of the vocalic *uh* variant (V) and the vocalic-nasal *uhm* variant (VN) in the speech of female and male speakers in the United States. They found a higher VN ratio in female speech across age groups. Similar findings have been reported for British English (Tottie, 2011) and German speakers (Belz, 2021).

In a broad corpus study of spoken and written data Wieling et al. (2016) investigated the use of FP variants in 6 languages (English, Dutch, German, Norwegian, Danish, Faroese). They found the VN variant to be more frequent in young, female speech across their selection of Germanic languages and interpret this as evidence for FPs as a socio-linguistic variable and an ongoing cross-linguistic language change lead by young female speakers. Fruehwald (2016) further develops this argumentation and places FPs within the linguistic system related to social variables and subject to language change. Next to the variation in form this prior research has also shown a difference in FP frequency across the same variables with male speakers producing more FPs than female speakers and older speakers more than younger speakers (Tottie, 2011, 2014).

In terms of their prosody, FPs have been described as having a relatively low fundamental frequency (F0) and a level or gradually falling F0 contour (Shriberg, 2001; Belz and Reichel, 2015). In languages like English, German and also Russian, prosodically non-prominent or unstressed syllables are phonetically reduced to central vowel nuclei and in the case of following nasals the vowels can even be deleted completely. This form of segmental reduction along with prosodic non-prominence results in an assumed lower salience in perception. FPs frequently show several of these phonetic qualities related to lower salience. In fact, listeners are not very good at detecting them in online tasks or estimating their frequency depending on their phonetic form. In a perception study Niebuhr and Fischer (2019) found filler particles to be less reliably estimated by listeners if produced shortly and nasally. That is, short *uhm*s are less salient than long *uh*s.

FPs' low salience favors an interpretation as a symptom of difficulties in speech planning (Maclay and Osgood, 1959; Berthold and Jameson, 1999; Shriberg, 2001). Therefore, FPs have been used as an indicator for reduced fluency in bilingual speech [for an overview and discussion see de Jong (2018)]. The increased use of FPs has been related to cognitive load rather than language fluency (Vasilescu and Adda-Decker, 2006). Monitoring two or more languages and selecting appropriate structures according to the context is a cognitively challenging task (Kroll and Gollan, 2014), which might be one of the reasons for increased FP use in bilingual speech.

This view of FPs is supported by the correlation between cognitive load and FP frequency (Berthold and Jameson, 1999; Bortfeld et al., 2001; Betz et al., 2023). These studies of cognitive load revealed a higher frequency with increased task difficulty either of symptoms of reduced output quality (e.g. false starts and repairs) or reduced output rate (e.g. articulation rate and pause frequency). As part of the latter category, a higher frequency of FPs can be observed in situations with higher cognitive load, i.e. higher demands on a person's working memory as a result of carrying out a difficult task or multiple tasks simultaneously. The increased frequency and duration of FPs can be interpreted as a strategy of the speaker signaling more speech is still to come during more efficient speech planning. This aspect of buying time in the use of FPs is not only relevant for the speaker and speech planning but also speech processing. There seems to be a beneficial effect of FPs on comprehension (FoxTree, 2001). Listeners can recall words more easily if they are preceded by FPs (FoxTree, 2001), and can recall and retell short story passages better if FPs are included in the input (Fraundorf and Watson, 2011). This has been found to be the case for both native and non-native listeners (Watanabe et al., 2005). The production of FPs is, therefore, not only speaker but also listener oriented and contributes to mutual comprehension.

The relation between FP frequency and cognitive load is in line with reports of increased FP frequency in contexts with an increased level of abstractness or complexity or in cases of situational uncertainty (Rochester, 1973; Betz et al., 2023). Higher use of disfluencies like FPs has also been reported in the speech of older speakers, which has been related to increased difficulty in lexical retrieval with increasing age (see Mortensen et al., 2006 for an overview of studies on age related aspects of disfluencies). Additionally, Tottie (2014) reports register as a factor in FP frequency: the more private and intimate a conversational situation, the fewer FPs are produced. The opposite holds for non-private and more formal contexts (see also Staley and Jucker, 2021).

While FPs are related to speech planning and processing, their occurrence has been reported to be rule-based at discourse or syntactic boundaries in spontaneous discourse (e.g., Swerts, 1998; Clark and FoxTree, 2002; Kjellmer, 2003; Belz, 2021). FPs are produced to signal delays in speech production and frequently introduce new topics or paragraphs (Clark and FoxTree, 2002) and share distributional and functional aspects with lexicalized discourse markers (Kjellmer, 2003; Schegloff, 2010; Knudsen et al., 2020). Both occur at discourse boundaries, indicating a shift on discourse level, i.e., the beginning of a new sequence or topic. They are used to structure discourse and also in turn management especially in cases of turn-holding. The analysis in this study considers the frequency of FPs and their forms and does not consider possible different functions of FPs.

FPs are language-specific in terms of their phonological structure and follow discourse syntax. They can therefore be seen as words in the respective language, as suggested by Clark and FoxTree (2002). Additionally, FPs show a relation to the social language use and language change. So, FPs and their use are one of many aspects of grammar learners have to acquire in a (second) language. However, since FPs tend not to be perceptually prominent, they may pose a challenge in language acquisition. As a phenomenon at the edge of our consciousness, they might be more difficult to learn than more salient aspects of language. This would be especially relevant in bilingual contexts with limited input.

### 1.2 Filler particles in bilingual speech

Investigations of bilingual speech and their use of FPs have shown deviances from monolingual FP use and provided insight into second language (L2) speech planning, phonology and discourse management. Since FPs can be related to speech planning, their frequency has been used as a measure of L2 fluency (de Jong et al., 2013; Lickley, 2015; de Jong, 2018; Belz and Odebrecht, 2022). Making this link is not unproblematic since FPs are not exclusively used by L2 speakers. Fluency has been defined as speaking “without (unnatural) hesitation” (de Jong, 2016, p.113). The importance of the baseline comparison, i.e. what constitutes an (un)natural hesitation, is therefore essential.

In a study on 18 monolingual and 52 bilingual speakers of Dutch with different first languages (L1s), Turkish and English de Jong (2016) investigated the frequency of FPs. De Jong's analysis shows higher use of FPs in L2 speakers who produce more pauses and FPs within utterances than L1 speakers. De Jong relates this to linguistic planning or micro planning, which takes more time or effort in L2 speech, leading to more hesitations. However, both speaker groups (L1 and L2) produce more FPs before lower frequency words, i.e. in contexts of more demanding lexical retrieval. The results of this study illustrate that when considering FP frequency that hesitating is natural in both L2 and L1 speech, yet there are differences in FP frequency within utterances and similarities in similarly challenging contexts.

In a corpus analysis of speech by advanced learners of L2 English with French L1, Gilquin (2008) found a higher use of FPs by the L2 speakers compared to monolingual English speakers, and argues that this results from the L2 speakers' underuse of lexical fillers and discourse markers in their L2. Additionally, the L2 speakers in the corpus produce relatively more V fillers, i.e., the VN-to-V ratio is lower in French-English bilinguals than in English monolinguals. Gilquin (2008) relates this to a possible transfer from French.

In a study on 15 female French-German simultaneous bilinguals Lo (2020) found further evidence for language influence in bilingual FP use. The simultaneous bilinguals in their study produced language-specific VN ratios which are higher in German and lower in French, as well as language-specific FP vowel quality. However, there is an effect of language dominance: German-dominant bilinguals produce fewer V variants than French-dominant bilinguals in both their languages.

Similarly, Muhlack (2023) looked ad FP realization in Spanish-English bilinguals and found language specific and contact specific patterns. Their analysis focused of 20 female speakers, 10 of them with Spanish L1 and 10 with English L1 and found a higher FP frequency in their respective L2. Additionally, their results showed a V preference in Spanish and in the L2 English of bilinguals with L1 Spanish and a VN preference in English and in the L2 Spanish of bilinguals with L1 English.

In a larger corpus study on Dutch-English bilinguals de Boer and Heeren (2020) looked at 58 female speakers with Dutch as L1 and English as L2. In their analysis of FPs they did not find an increased use of FPs in the L2 in this group of university students. There was a difference across the two languages regarding FP type: In Dutch the VN form was less frequent than in the English.

These studies show that bilinguals tend to produce more FPs in their L2, yet, not all bilingual groups show the same behavior. In the language pairs these studies addressed one favored the V form (French, Spanish, Dutch) while the other favored the VN form (German, English). This language specific pattern is partly produced by bilingual speakers, yet also influences the use of FP form in their respective other language. These studies did not, however, include monolingual data for comparison and also only focused on one gender. Whether or not there are differences between mono- and bilingual speakers as well as differences across genders in bilingual speech remains to be addressed.

The distribution of FPs also seems to be language- or culture-specific. In a cross-linguistic corpus study, Rose and Watanabe (2019) compared the use and duration of silent pauses and FPs in unscripted speech of English and Japanese speakers. In their study they investigated the *pause filler hypothesis*, i.e. whether FPs are produced to fill a silence longer than a certain threshold. In their corpus data they found no difference between languages in terms of silence and FP duration within utterances. However, there were differences in pause duration at utterance boundaries: Japanese speakers produced longer silences before FPs as well as longer silences overall compared to English speakers. This tolerance for longer silences was also transferred to their L2 English productions. The findings of this study are in line with prior observations that Japanese speakers along with speakers of other Asian languages have higher tolerance for silences than speakers of e.g. English (Rose and Watanabe, 2019). This is evidence for the language-specific use of FPs in languages like English and Japanese, as well as for transfer of these usage patterns depending on the context from the L1 to the L2.

### 1.3 Bilingual heritage speakers

Prior research on FPs has included a variety of bilingual speaker groups from foreign language learners to simultaneous bilinguals. While the area of bilingualism covers a wide spectrum this study focuses on bilingual heritage speakers. Heritage speakers (HSs) pose a specific case of bilinguals of a minority or heritage language and a majority language. The majority language (ML) is typically used in most areas of the public sphere, e.g. work and education, while the heritage language (HL) as a minority language in the larger society is acquired in specific contexts and typically spoken at home, e.g. with relatives and friends (Montrul, 2015; Polinsky, 2018).

The language acquisition of these bilinguals is characterized by early or simultaneous bilingualism, limited or specialized input and use in the HL, and a hierarchical relationship in societal status between the languages. The use of the HL is limited to certain interlocutors, genres and communicative situations. Prior research on HLs has reported language contact phenomena like code switching and calquing, as well as the emergence of new linguistic structures possibly leading to language change (Muysken, 2013). A growing body of research suggests HSs could be a link between second language learners and monolingually raised speakers on a native-speaker continuum (Wiese et al., 2022, e.g.).

The nature of HSs' language acquisition with the HL spoken in informal contexts at home and the ML spoken in more areas and contexts of everyday life can explain these speakers' variation in areas of pragmatics and the lexicon. Linguistic structures at the syntax-pragmatics interface are assumed to be more vulnerable in language contact compared to structures at the syntax-lexicon interface (Sorace, 2011). Additionally, the specifics of pragmatics are closely intertwined with cultural norms and social practices (e.g. V(ous)-T(u) contrast in languages like French used to indicate level of formality, politeness and intimacy). Living in a different country from the homeland of the respective language gives HSs little possibility to experience and acquire cultural aspects of language use including social group variables and restricts or specializes them to informal interactions with family members (Polinsky, 2018).

As with variation and culturally specific ways of language use, the HSs' lexicon is defined by the type and amount of input. HSs easily master informal vocabulary describing everyday events parallel to the kind of opportunities for language use they are presented with (Polinsky, 2018). This again is linked to their relationship with HL interlocutors in a familial setting and their cultural context of HL acquisition in a different country. Therefore, subtleties in lexical choice deviate from monolingual norms as do the pragmatic aspects of language use.

It is important to note that deviances in the HL usually go hand in hand with native competences in the ML. However, there are some studies that suggest a bidirectional influence of both languages, (e.g., van Rijswijk et al., 2017) and general differences in bilingual language processing compared to monolingual processing (Kroll and Gollan, 2014; Korenar et al., 2023). In their study covering different language contact situations including several HLs and MLs, Wiese et al. (2022) demonstrate that not all deviances found in HL research can or should be related to effects of bilingualism. They report on deviances from linguistic norms based on standard varieties as reported in the literature by both monolingually and bilingually raised speakers of different languages in different countries (Germany and the United States). They find that deviances are most present in informal language, which is the dominant register for most HSs. The standard variety usually needs to be studied in a formal educational context, not necessarily available to HSs. Wiese et al. (2022) argue for a native speaker continuum and also promote investigating register variety in HL research when comparing language productions by HSs to a baseline of e.g., monolingual speakers.

Heritage speakers are neither L2 learners nor monolingually raised native speakers. As such they represent a connecting link in bilingual research on the native speaker continuum. The specific aspects of their language contact situation results in language contact phenomena. While the area of morpho-syntax is well studied in several languages, the phonetics of HSs' speech as well as the specifics of social pragmatic behavior are not well represented in HL research [for an overview see Polinsky (2018)]. The use of FPs is connected to both areas of research. Therefore, this study addresses the frequency and segmental form of FPs as well as their use in the speech of HSs in both their languages.

### 1.4 Research questions and hypotheses

This study investigates the frequency and use of FPs in the speech of monolingual and bilingual speakers of the MLs English and German and their HL Russian as well as HL German in the United States. In the first analysis FPs are investigated as symptoms of speech planning and cognitive load as discussed in Section 1.1. The second analysis treats FPs as lexical items of the respective languages. If FPs have word status, the HSs should acquire them in both languages with representations of both forms in their lexicon. Therefore, the objective of this study is to look for evidence for the language-specific form and use of FPs. As native speakers of two languages, HSs are expected to use distinct filler particle forms in the different languages. Additionally, the second analysis addresses the variation in FP use related to the social variables gender and age which has been reported for the MLs English and German. Since HSs are part of a different socio-linguistic environment compared to monolingual homeland speakers HSs might differ in the use of FP forms. In more detail the research questions and hypotheses are:

1. Do both mono- and bilingual speakers increase the number of FPs in contexts with assumed higher pressure on speech planning related to cognitive load?
  H1 a: Heritage speakers produce more FPs compared to monolingual speakers in the respective language overall due to higher cognitive effort monitoring two languages.
  H1 b: Monolinguals mirror this effect in situations with more pressure on language form (e.g. in formal register) and in an age related effect.
2. Do heritage speakers produce language specific FPs forms in their two languages? Can the use of different segmental forms of FPs be related to socio-linguistic aspects reported for the MLs of the HS?
  H2 a: The languages English, German and Russian show different filler particle form preferences, i.e. VN filler ratios.
  H2 b: The use of filler particle form can be related to socio-linguistic aspects of gender and age group across languages.
  H2 c: Heritage speakers distinguish the use of FP variants in their two languages.

## 2 Method

### 2.1 The RUEG corpus

The research questions presented in Section 1.4 are investigated using data taken from the RUEG corpus (Wiese et al., 2021), a corpus of spontaneous speech including data of both mono- and bilingual speakers of different age groups (adolescent: 14–18 years; adult: 22–35 years). The data in the RUEG corpus comprise recordings of Greek, Russian, Turkish and German heritage speakers in Germany and the U.S. Additionally, monolingual data in all five languages were elicited. The corpus data is transcribed and annotated on several layers including parts of speech. For this study English, Russian and German data from version RUEG 1.0 SNAPSHOT was used^1^. The corpus version contains 4468 narrations by 736 speakers with 326 monolingual and 412 bilingual speakers. The unified method of the corpus allows for high cross-linguistic comparability to investigate language contact phenomena as well as intra-individual comparison of bilingual speakers. The narrations within the RUEG corpus were elicited by means of a video depicting a car accident, following the Language Situation Method (Wiese, 2020). The video of the car accident was used to prompt participants to explain what happened in two situations (formal vs. informal) and two modes (written vs. spoken). Participants were asked to provide a police report both in written form as well as in form of a voice message on the phone. This is referred to as the formal situation. Participants were also asked to describe the incident to a friend by means of a text message as well as a spoken voice message yielding an informal situation. The procedure was designed involving two elicitors yielding a “one person, one language” or rather “one person, one language register” scenario for the formality of the situation.

To address the research questions in this study data from the following speaker groups are considered: majority English speakers with Russian heritage, majority English speakers with German heritage, as well as majority German speakers with Russian heritage, in both their languages. Additionally, data from monolingual English speakers, monolingual German speakers and monolingual Russian speakers are considered. An overview of the number of speakers per speaker group can be found in Table 1.

**Table 1:** Mean age and age of onset (AoO) of the ML along with number of speakers per speaker group and age group.

**Speaker group**	**Age group**	**Mean age**	**Mean AoO**	**No of speakers**
German monolinguals	Adolescent	16.8	0	33
	Adult	22.1	0	31
German HSs in the U.S.	Adolescent	15.0	0.3	29
	Adult	25.1	1.0	7
Russian HSs in Germany	Adolescent	15.2	0.8	29
	Adult	22.9	1.3	34
Russian monolinguals	Adolescent	16.5	0	34
	Adult	26.1	0	33
Russian HSs in the U.S.	Adolescent	15.3	2.2	35
	Adult	26.7	3.6	33
English monolinguals	Adolescent	15.5	0	29
	Adult	26.5	0	32

### 2.2 Queries and data treatment

For the frequency analysis, FPs were extracted as transcribed in the different languages from the English, German and Russian sub-corpora based on the written transcript. The included FP forms for all languages are presented in Table 2.

**Table 2:** Different FP transcriptions within the English, German and Russian sub-corpus of the RUEG corpus.

**Language**	**Filler particle**
English	eh / er [ə:], uh / ah [ɐ:], em / ehm [əm], uhm [ɐm], mm [m]
German	äh [ɛ:], ähm [ɛ:m], öh [ø:], öhm [øm], hm / mh [m]
Russian	э[ɛ], a [a:], эм [ɛm], aм [a:m], хм [xm], м [m]

Queries were based on the transcriptions and included possible transcriptions of lengthening, since not all language sub-corpora included part of speech labels for items like FPs and discourse markers. The corpus queries were therefore language-dependent and are reported in the Supplementary Material. Queries were carried out in ANNIS (Krause and Zeldes, 2016). For the Russian FP *a* the instances in the corpus were checked for possible overlap with conjunctions which were then excluded from the analysis. For the English and German data, only narrations by monolingual speakers or bilingual speakers of either Russian or German as a HL were included in the current analysis. The corpus search for the remaining FP candidates resulted in 4,082 FPs produced in 371 Russian narrations, 2,212 FPs in 293 English narrations and 3294 FPs in 304 German narrations.

The FP frequency was normalized per 100 words within each narration. The language-specific FP forms were categorized as either vocalic (V), nasal (N), vocalic-nasal (VN) or consonant-nasal (CN) depending on the segmental structure. Vowel quality was not taken into consideration in the current analysis. The data comprises two age groups comparing adolescents (mean age: 15.6) and adults (mean age: 24.9). Table 1 gives an overview of the different speaker groups, the number of speakers, their mean age and mean number of FPs per 100 words as well as mean age of onset (AoO) for the bilingual speakers. Statistical analysis were run in R (R Core Team, 2023), RStudio (Posit team, 2023) using the lmer() function from the lme4 package (Bates et al., 2015), the step() function from the lmerTest package (Kuznetsova et al., 2017) for finding the best fitting model and the emmeans() function from the emmeans package (Lenth, 2022) for *post-hoc* testing. Figures were created using ggplot2 (Wickham, 2016).

## 3 Results

### 3.1 Filler particle frequency across speaker groups

The mean FP ratio for the whole data set was 7.1 FPs per 100 words. There is a difference in FP ratio across the three languages under investigation. The mean FP ratio is highest for the Russian data (n = 4 082, $\bar{x}$= 9.56) followed by German (n = 3 294, $\bar{x}$= 6.15) and English (n = 2 212, $\bar{x}$= 5.24). These values include both mono- and bilingual speakers within each language.

**Figure 1:**
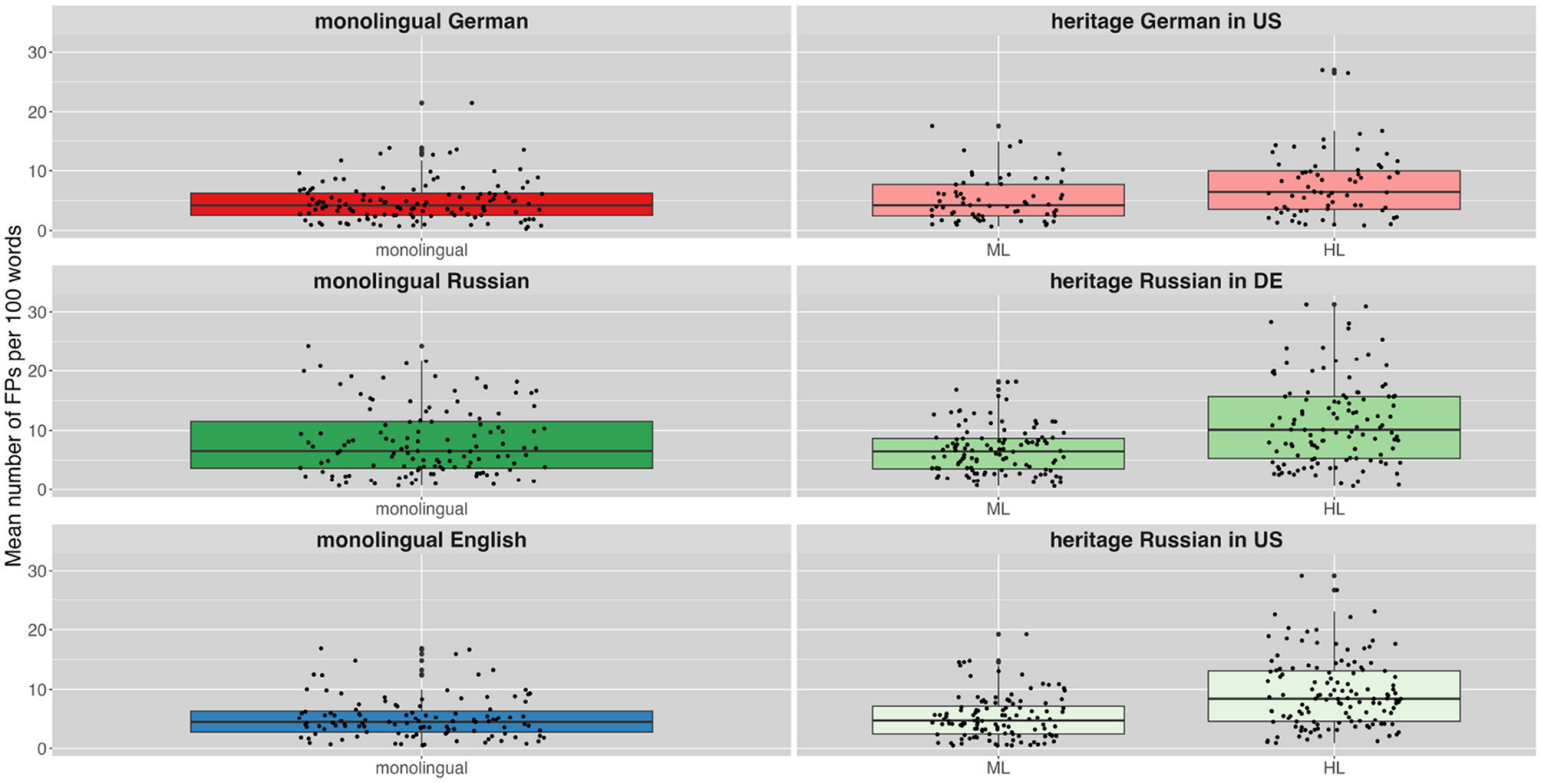
Mean ratio of filler particles produced per 100 words by different speaker groups in their different languages (ML, majority language; HL, heritage language).

Figure 1 gives an overview of FP ratio across languages and speaker groups along with the individual variation. The monolingual speakers' FP ratios are presented in the left column and the bilingual heritage speakers' ratios on the right. The colors indicate the different speaker groups. For heritage speakers the majority language of the respective country (German for HSs in Germany, English for HSs in the U.S.) is indicated as ML and their heritage language as HL. Bilinguals of all language combinations produced a lower FP ratio in their ML language (US_D: $\bar{x}$= 5.19, US_R: $\bar{x}$= 5.34, DE_R: $\bar{x}$= 6.69) than in their HL language (US_D: $\bar{x}$= 7.64, US_R: $\bar{x}$= 9.41, DE_R: $\bar{x}$= 11.28). Table 3 provides a more detailed overview of the number and ratios of filler particles across languages and speaker groups.

**Table 3:** Distribution of FPs and FP variant across languages and speaker groups.

**Language**	**Speaker group**	**Nr of FPs**	**FP%**	**Nr of VN**	**VN %**
German	German monolinguals	1124	4.86	692	57.00
	German HS in US	694	7.64	403	52.00
	Russian HS in DE	1476	6.69	870	55.00
Russian	Russian monolinguals	912	8.13	85	10.00
	Russian HS in DE	1823	11.20	865	45.00
	Russian HS in US	1340	9.22	412	29.00
English	English monolinguals	723	5.18	478	67.00
	German HS in US	489	5.19	308	64.00
	Russian HS in US	994	5.34	470	54.00

Mean number of FPs calculated per 100 words (FP%); Mean number of VN variants calculated per 100 FPs (VN%).

**Figure 2:**
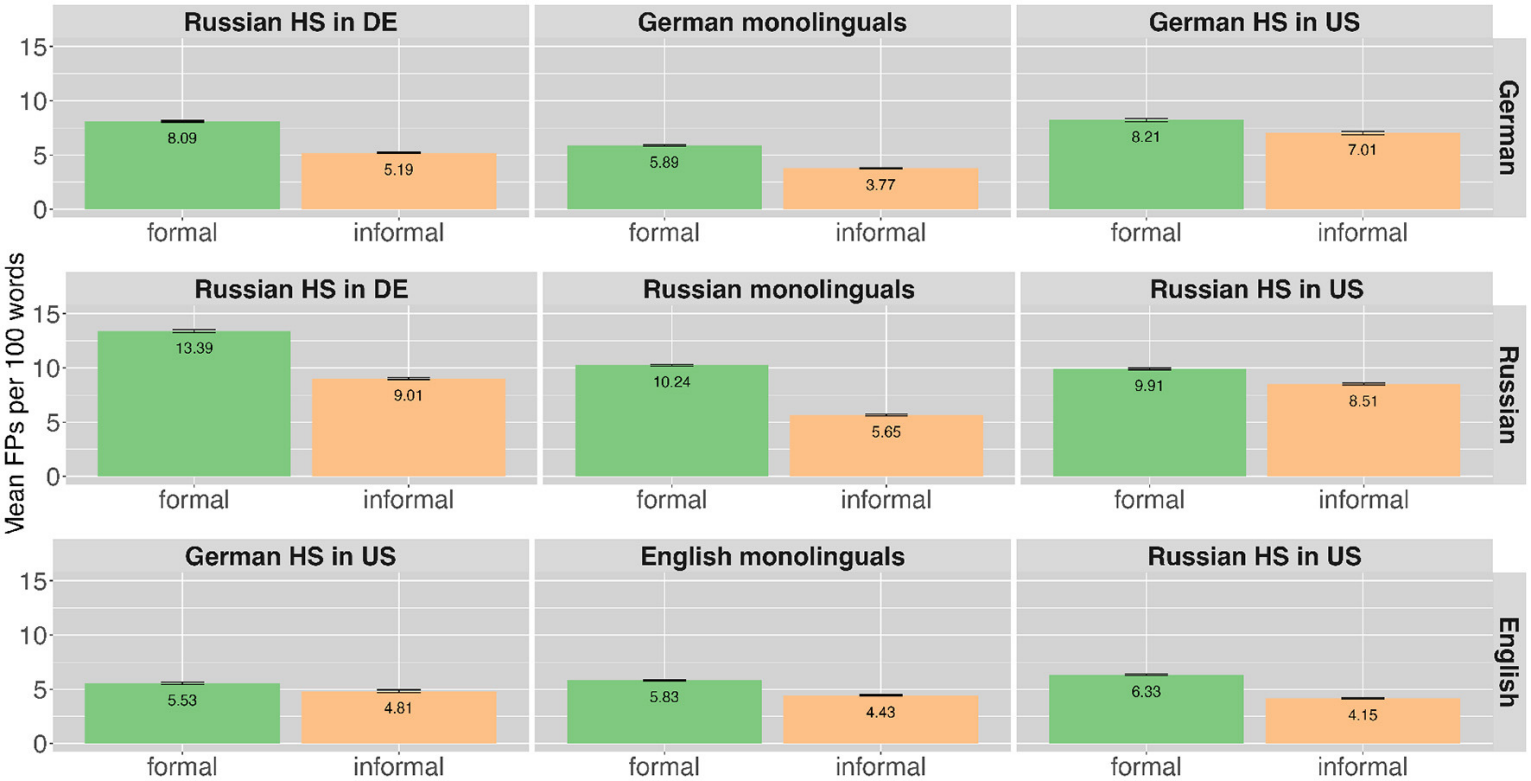
Mean ratio of filler particles produced per 100 words by different speaker groups in the different languages (in rows) across the different formalities along with error bars.

To check for aspects related to assumed higher cognitive load that also apply to monolingual speakers the two variables formality of the situation and age group were included in the analysis. Overall the FP frequency was higher in the formal compared to the informal situations in all languages (German formal: *n* = 2,150, 7.22%; German informal: *n* = 1,144, 5.00%; Russian formal: ***n*** = 2,519, 11.07%; Russian informal: *n* = 1,556, 7.76%; English formal: *n* = 1,484, 5.97%; English informal: ***n*** = 722, 4.41%). Figure 2 presents FP ratios across speaker groups, languages and the two levels of formality. The monolingual speaker groups are presented in the middle column of the figure and the bilingual heritage speakers on both sides in the respective language. FP ratios are presented as produced in the respective languages: German productions in the top row, Russian productions in the middle and English productions in the bottom row. That is, there are two panels for each heritage speaker group, one in each language. As can be seen in the higher green bars in Figure 2 the number of FPs produced by speakers is higher in formal compared to informal situations. This is true for all speaker groups and languages. Table 4, additionally, gives a more detailed overview of FP occurrence and normalized frequency across languages, speaker groups and situations.

**Table 4:** Distribution of FPs and FP variant across languages, speaker groups, and situations.

**Language**	**Speaker group**	**Situation**	**Nr of FPs**	**FP%**	**Nr of VN**	**VN %**
German	German monolinguals	Formal	767	5.89	485	65.00
		Informal	357	3.77	207	50.00
	German HS in US	Formal	430	8.21	256	53.00
		Informal	264	7.01	147	51.00
	Russian HS in DE	Formal	953	8.09	569	58.00
		Informal	523	5.19	301	52.00
Russian	Russian monolinguals	Formal	639	10.24	62	9.00
		Informal	273	5.65	23	12.00
	Russian HS in DE	Formal	1115	13.39	554	49.00
		Informal	708	9.01	311	40.00
	Russian HS in US	Formal	765	9.91	232	31.00
		Informal	575	8.51	180	26.00
English	English monolinguals	Formal	489	5.83	335	67.00
		Informal	234	4.43	143	67.00
	German HS in US	Formal	313	5.53	207	66.00
		Informal	176	4.81	101	61.00
	Russian HS in US	Formal	682	6.33	317	54.00
		Informal	312	4.15	153	54.00

Mean number of FPs calculated per 100 words (FP%); Mean number of VN variants calculated per 100 FPs (VN%).

**Figure 3:**
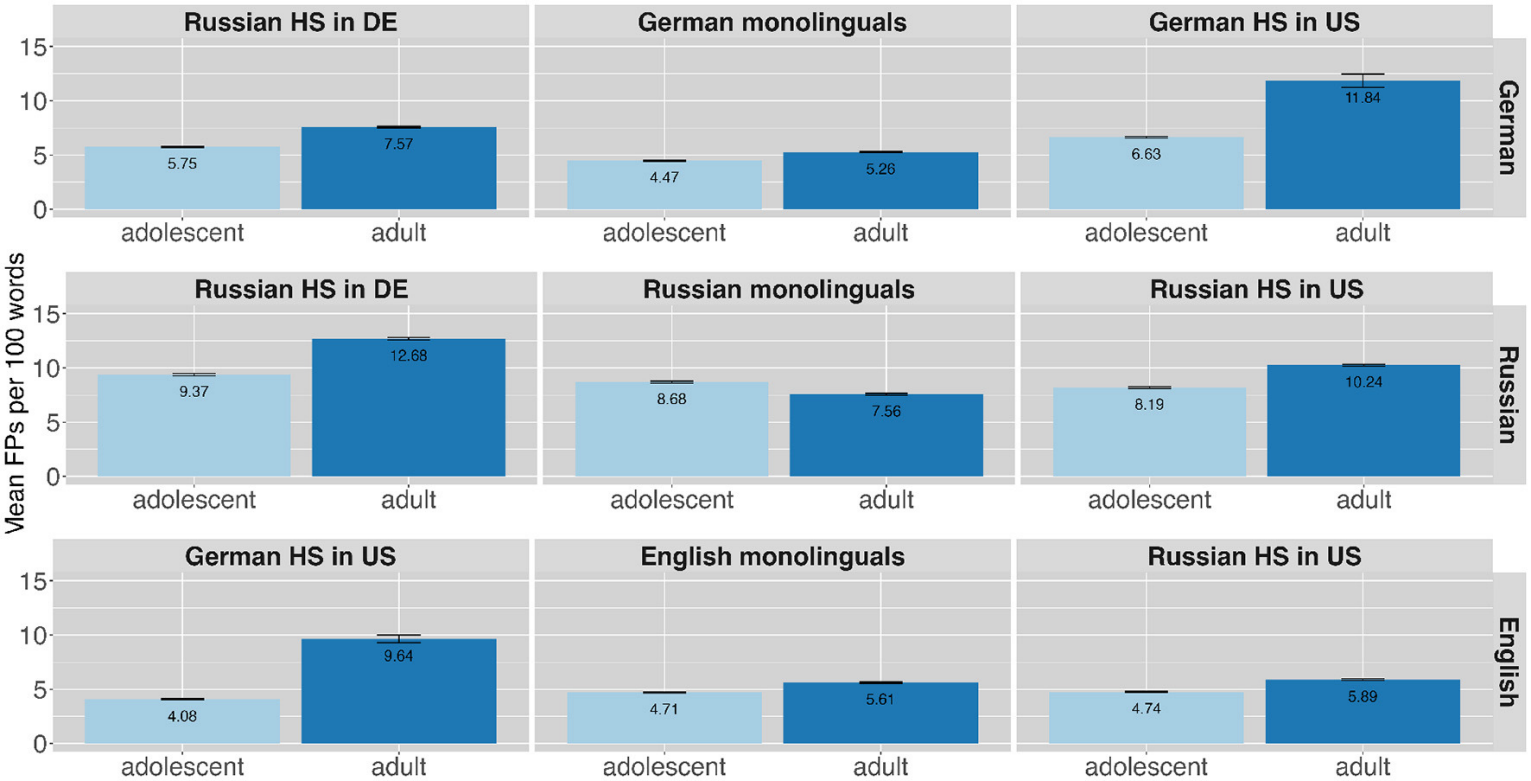
Mean ratio of filler particles produced per 100 words by different speaker groups in the different languages (in rows) across the different age groups along with error bars.

The second aspect related to assumed higher cognitive load is age. And indeed, overall the FP frequency was higher in the adult compared to the adolescent speaker group in all languages (German adult: *n* = 1,721, 6.92%; German adolescent: *n* = 1,573, 5.55%; Russian adult: *n* = 2384, 10.20%; Russian adolescent: *n* = 1,691, 8.70%; English adult: ***n*** = 1,171, 6.13%; English adolescent: *n* = 1,035, 4.51%). Figure 3 shows the FP ratios across speaker groups and languages for the two age groups. The plots are arranged as in Figure 2. The higher dark blue bars indicate higher use of FPs for the adult compared to adolescent bilingual speakers in all languages and language pairs, while there seem to be less differences between age groups in monolingual speakers. Table 5 gives an overview of the numbers by language, speaker group and age group. The comparison of the mean FP ratios across the different age groups shows that adults produce more FPs than adolescents across all languages and speaker groups.

**Table 5:** Distribution of FPs and FP variant across languages, speaker groups, and age groups.

**Language**	**Speaker group**	**Age group**	**Nr of FPs**	**FP%**	**Nr of VN**	**VN %**
German	German monolinguals	Adolescent	510	4.47	289	57.00
		Adult	614	5.26	403	58.00
	German HS in US	Adolescent	494	6.63	283	53.00
		Adult	200	11.84	120	50.00
	Russian HS in DE	Adolescent	569	5.75	343	57.00
		Adult	907	7.57	527	53.00
Russian	Russian monolinguals	Adolescent	463	8.68	57	15.00
		Adult	449	7.56	28	6.00
	Russian HS in DE	Adolescent	623	9.37	299	47.00
		Adult	1200	12.68	566	43.00
	Russian HS in US	Adolescent	605	8.19	222	33.00
		Adult	735	10.24	190	25.00
English	English monolinguals	Adolescent	269	4.71	165	62.00
		Adult	454	5.61	313	72.00
	German HS in US	Adolescent	305	4.08	210	67.00
		Adult	184	9.64	98	49.00
	Russian HS in US	Adolescent	461	4.74	233	58.00
		Adult	533	5.89	237	51.00

Mean number of FPs calculated per 100 words (FP%); Mean number of VN variants calculated per 100 FPs (VN%).

**Figure 4:**
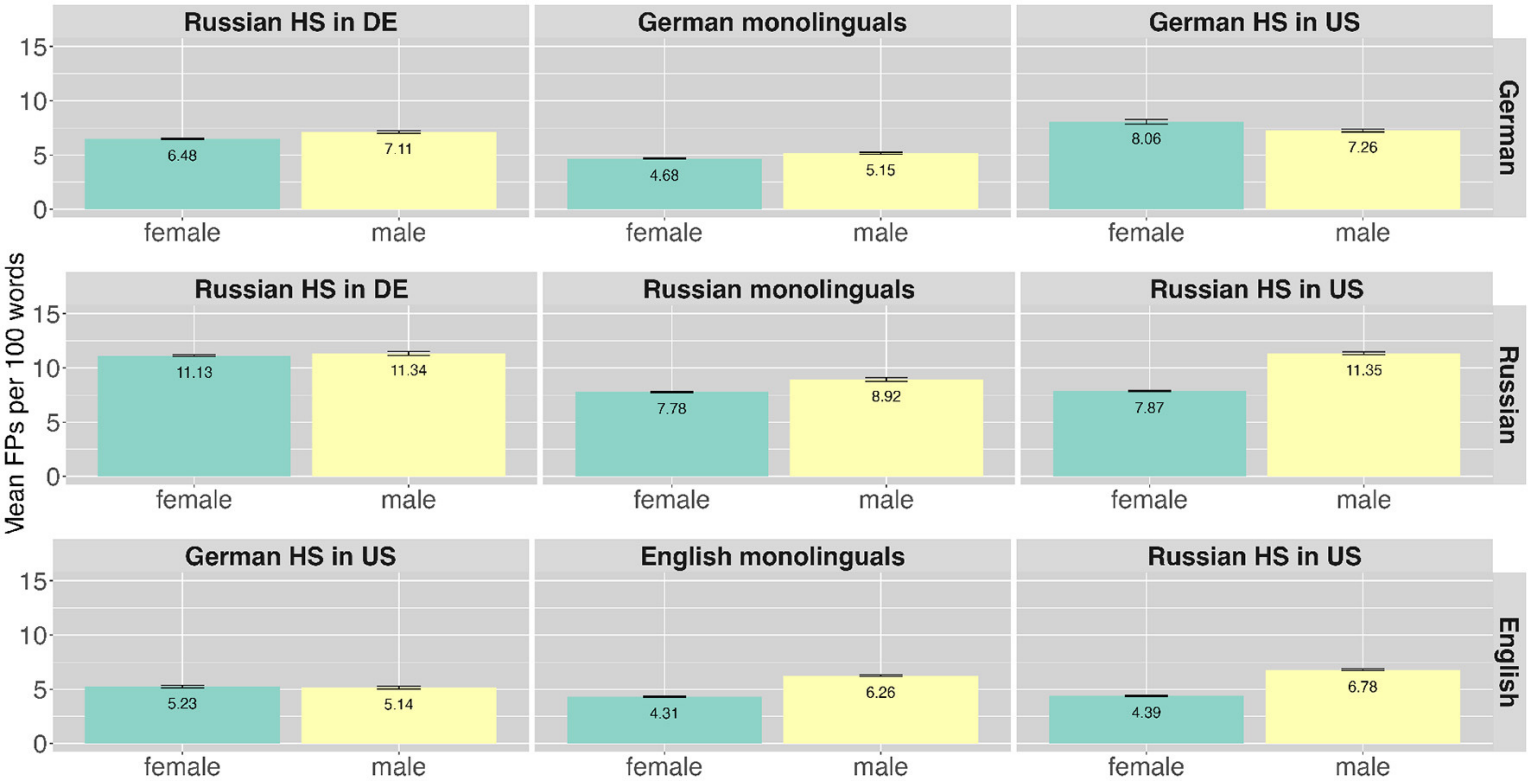
Mean ratio of filler particles produced per 100 words by different speaker groups in the different languages (in rows) across the different genders along with error bars.

Next to speech planning effort the aspect of FP frequency has also found to be related to the socio-linguistic parameter gender. Part of the frequency analysis, therefore also includes gender. Overall the FP frequency was higher in male compared to female speakers across all languages ^2^ (German male: *n* = 1,405, 6.39%; German female: *n* = 1,889, 5.99%; Russian male: *n* = 1,438, 10.61%; Russian female: *n* = 2,637, 8.89%; English male: *n* = 1,133, 6.17%; English female: *n* = 1073, 4.53%). Figure 4 shows the FP ratios across speaker groups and languages for the two genders. The plots are arranged as in Figure 2. The higher yellow bars indicate higher use of FPs for the male compared to female speakers in all languages and language pairs. Table 6 gives an overview of the numbers by language, speaker group and gender. The comparison of the mean FP ratios across the two genders shows that male speakers produce more FPs than female speakers across all languages and most speaker groups with the exception of Russian HSs in Germany in their HL and German HSs in the U.S. in their ML.

**Table 6:** Distribution of FPs and FP variant across languages, speaker groups, and genders.

**Language**	**Speaker group**	**Gender**	**Nr of FPs**	**FP%**	**Nr of VN**	**VN %**
German	German monolinguals	Female	658	4.68	471	66.00
		Male	466	5.15	221	43.00
	German HS in US	Female	318	8.06	214	69.00
		Male	376	7.26	189	37.00
	Russian HS in DE	Female	913	6.48	577	61.00
		Male	563	7.11	293	43.00
Russian	Russian monolinguals	Female	619	7.78	70	12.00
		Male	293	8.92	15	7.00
	Russian HS in DE	Female	1276	11.13	597	44.00
		Male	547	11.34	268	46.00
	Russian HS in US	Female	742	7.87	264	31.00
		Male	598	11.35	148	25.00
English	English monolinguals	Female	322	4.31	267	79.00
		Male	401	6.26	211	52.00
	German HS in US	Female	245	5.23	185	77.00
		Male	244	5.14	123	50.00
	Russian HS in US	Female	506	4.39	276	61.00
		Male	488	6.78	194	43.00

A linear mixed regression was used to test if *language, bilingualism, formality of the situation, age group* and *gender* as well as the random intercept of the *individual speaker* and the random slope of the speaker dependent variable *gender* significantly predicted FP frequency. A model including random slopes of the speaker dependent variables *bilingualism, language* and *age group* did not converge and a model. We present the full model syntax of the model with the best fit we used in the Supplementary Material. The model revealed significant main effects of *language, bilingualism, formality of the situation, age group* and *gender* as well as an interaction of *language, situation* and *bilingualism, age group* (p < 0.001, conditional R^2^ = 0.50, marginal R^2^ = 0.31). A regression Table of the type III analysis of variance using the Satterthwaite's method is presented in Table 7.

**Table 7:** Type III analysis of variance table with Satterthwaite's method for FP frequency model.

	**Sum Sq**	**Mean Sq**	**NumDF**	**DenDF**	**F value**	**Pr(>F)**
Language	1288.12	644.06	2.00	634.43	51.92	0.0000
Bilingual	183.40	183.40	1.00	355.31	14.79	0.0001
Situation	1524.83	1524.83	1.00	629.31	122.93	0.0000
Gender	128.26	128.26	1.00	262.99	10.34	0.0015
Age_group	159.60	159.60	1.00	353.49	12.87	0.0004
Language:bilingual	65.34	32.67	2.00	638.83	2.63	0.0726
Language:situation	185.76	92.88	2.00	629.26	7.49	0.0006
Bilingual:situation	13.63	13.63	1.00	629.34	1.10	0.2950
Language:gender	14.12	7.06	2.00	872.16	0.57	0.5663
Language:age_group	12.09	6.04	2.00	928.59	0.49	0.6145
Bilingual:age_group	98.52	98.52	1.00	352.44	7.94	0.0051
Language:bilingual:situation	69.43	34.72	2.00	629.31	2.80	0.0616

*Post-hoc* Tukey pairwise comparisons for the interaction *language* and *situation* revealed a lower FP ratio in German and English compared to Russian for both formalities respectively (German formal: β = −4.1, SE = 0.5, df = 877.6, t = −8.3, *p* < 0.001; German informal: β = −2.5, SE = 0.5, df = 896.5, t = −4.9, *p* < 0.001; English formal: β = −5.0, SE = 0.5, df = 878.6, t = −10.0, *p* < 0.001; English informal: β = −2.8, SE = 0.5, df = 903.4, *t* = −5.3, *p* < 0.001). Additionally the FP ratio is higher in formal compared to informal narrations across languages respectively (German formal: β = 2.3, SE = 0.4, df = 615.9, *t* = 5.6, *p* < 0.001; English formal: β = 1.7, SE = 0.4, df = 633.7, *t* = 4.0, *p* < 0.001; Russian formal: β = 3.8, SE = 0.4, df = 631.1, *t* = 9.9, *p* < 0.001).

For the interaction *bilingualism* and *age group* the *post-hoc* Tukey pairwise comparisons revealed lower FP ratios in monolingual adolescents and adults compared to bilingual adults (monolingual adolescents: β = −3.0, SE = 0.6, df = 346.8, *t* = −5.1, *p* < 0.001, monolingual adults: β = −2.7, SE = 0.6, df = 338.9, *t* = −4.6, *p* < 0.001) as well as in bilingual adolescents compared to bilingual adults (bilingual adolescents: β = −2.6, SE = 0.6, df = 282.0, *t* = −4.6, *p* < 0.001).

Additionally, for the main effect of gender *post-hoc* Tukey pairwise comparisons revealed the FP ratio is lower in female compared to male speech (female: β = −1.4, SE = 0.4, df = 267.7, t = −3.2, *p* < 0.001).

The analysis of the normalized FP frequency reveals a language-specific FP ratio modulated by formality of the situation. FP frequency seems to be higher overall in the Russian data across formalities and a higher FP ratio in formal compared to informal speech for all languages. Additionally, FP frequency in our data is related to bilingualism modulated by age group. FP frequency in our data was higher for bilingual compared to monolingual speech in the case of adult heritage speakers. Additionally, a difference between age groups and two genders emerged with higher FP frequency in adult and male speakers.

### 3.2 Filler particle form across languages and genders

The most frequent FP form in the whole data set was the vocalic variant (V: *n* = 4,777, 50%) shortly followed by the vocalic-nasal variant (VN: *n* = 4,583, 48%). Since the number of FPs produced by speaker per narration varies, the FP form categories were normalized by the number of FPs occurring within each narration. There is a difference across languages, with VN being the predominant FP form in English (*n* = 1,256, 61%) and German (n = 1965, 55%), while being second to the V variant in the Russian data (V: *n* = 2,559, 69%, VN: *n* = 1,162, 22%). The nasal variant was not present in the English data and made up only a small percentage of the German (*n* = 58, 3%) and the Russian data (*n* = 146, 4%). The consonant-nasal filler only occurred in the Russian data with only 15 tokens found (0.5 %). Due to the low numbers of the nasal and consonant-nasal variants, the remaining analysis focuses on the more frequent VN and V forms.

**Figure 5:**
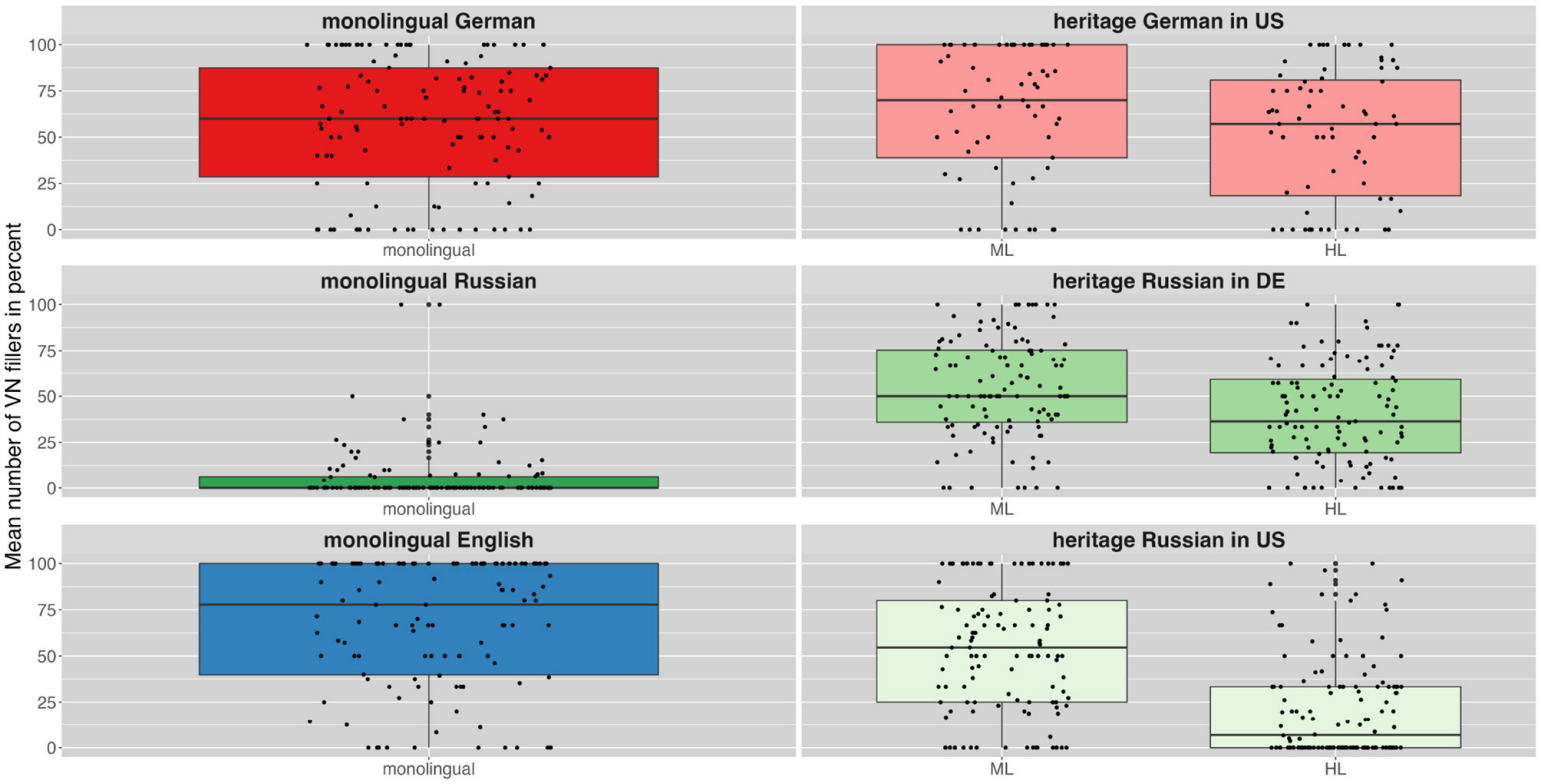
Mean ratio of VN filler particles produced per 100 fillers by different speaker groups in their different languages (ML, majority language; HL, heritage language).

The analysis will focus on the VN ratio calculated in relation to the overall number of FPs produced by the same speaker in the same narration. The V ratio can be approximated inversely. The ratio of VN fillers is lowest for monolingual Russian speakers (*n* = 52, 6%). The bilingual Russian speakers produce higher VN ratios in their Russian productions, Russian bilingual speakers living in Germany more so (*n* = 792, 40%) than bilinguals in the U.S. (*n* = 318, 20.8%). The rather high number of VN candidates in the Russian data set can, therefore, be attributed to the bilingual speakers' productions. The normalized VN frequency in the other languages and speaker groups is higher than (50%). This is also true for the bilingual speakers. A distribution of VN ratios across speaker groups can be seen Table 3 and in Figure 5. Monolingual speaker groups are again presented in the left column and bilingual heritage speaker on the right. The colors indicate the different speaker groups as in Figure 1. For heritage speakers the majority language of the respective country (German for HSs in Germany, English for HSs in the U.S.) is indicated as ML and their heritage language as HL. As can be seen, the monolinguals show different VN ratios across the three languages and the bilinguals show different VN ratios in their HL and ML respectively.

**Figure 6:**
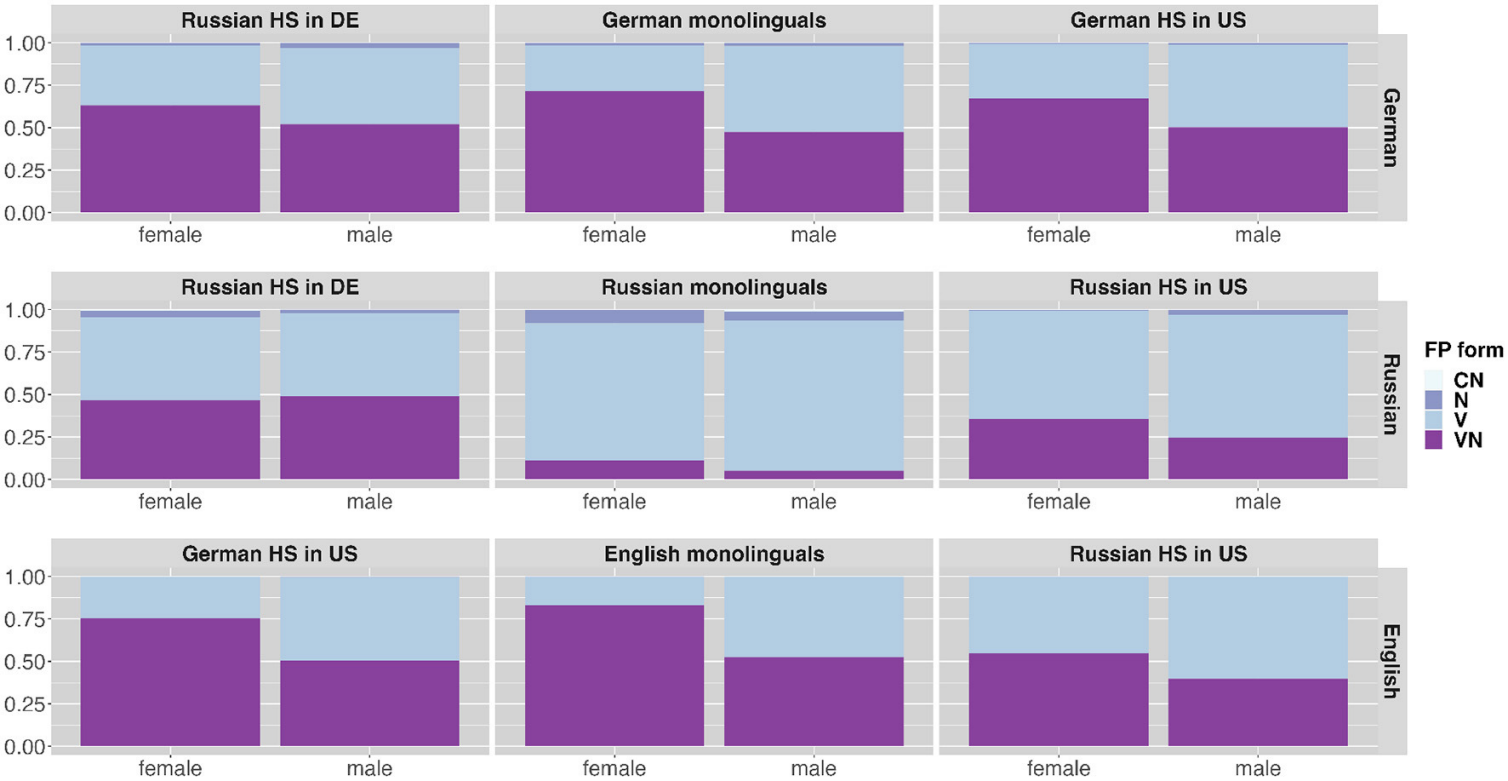
Distribution of FP forms (CN, consonant-nasal; N, nasal; V, vocalic; VN, vocalic-nasal) across different speaker groups in the different languages (presented in rows) across two genders.

**Figure 7:**
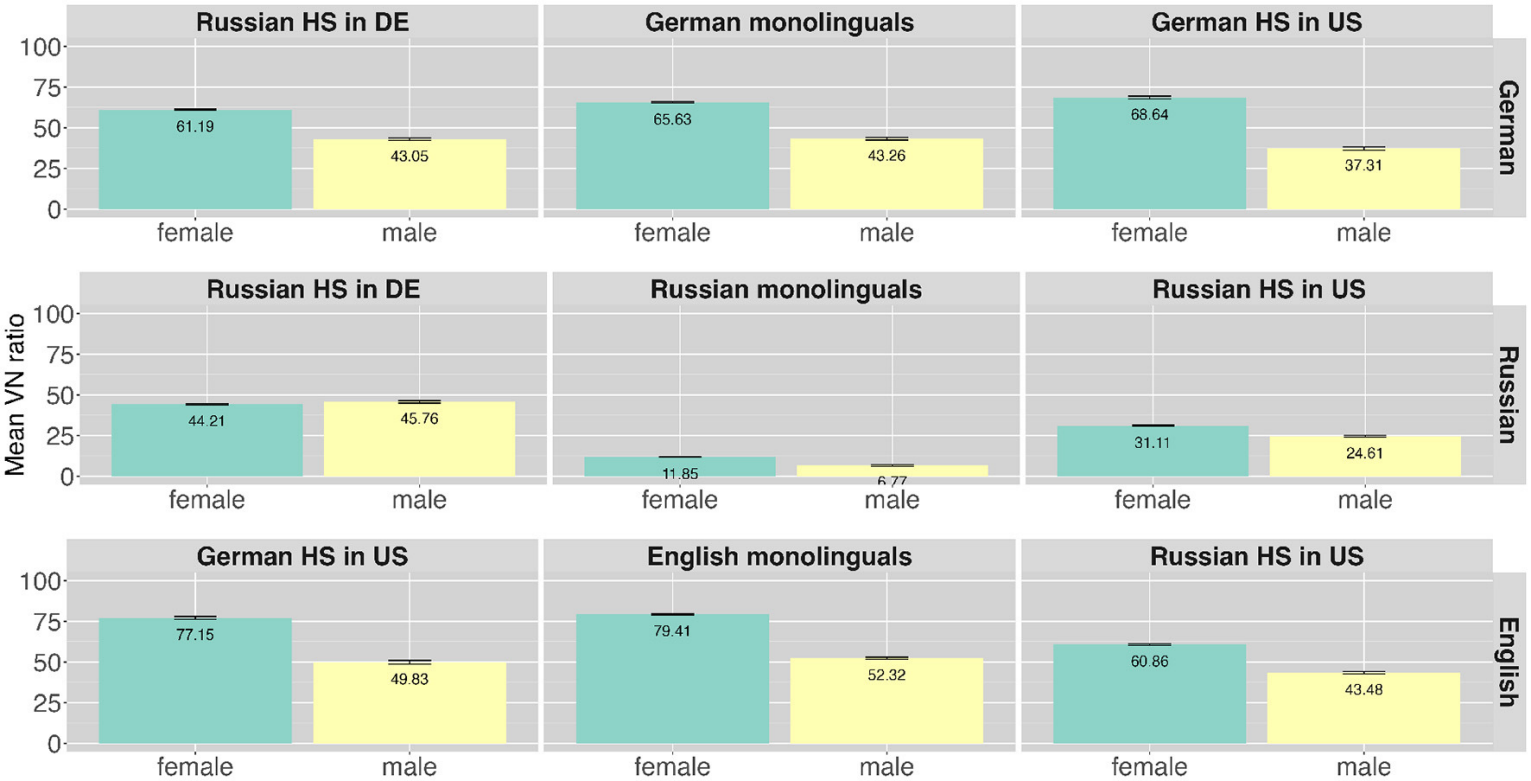
Distribution of VN variants across different speaker groups in the different languages (presented in rows) across two genders.

A difference in VN ratio can also be observed comparing their use in female and male speakers' narrations. In both languages, English and German female speakers produce higher VN rations (German: ***n*** = 1,262, 64%; English: *n* = 728, 71%) than male speakers (German: *n* = 703, 41%; English: *n* = 528, 49%). This difference is not present in the Russian data (female: *n* = 779, 22%; male: 383, 22%). The bilingual speakers in each language produce similar distributional patterns of FP forms related to gender compared to the monolingual speakers. Table 6 gives an overview of VN ratios for languages and speaker groups across two genders. Figure 6 presents the distribution of the different FP forms produced by the speaker groups in the three languages across two genders. The monolingual speaker groups are presented in the middle column of the figure and the bilingual heritage speakers on both sides in the respective language. Different FP forms are presented as produced in the respective languages: German productions in the top row, Russian productions in the middle and English productions in the bottom row. That is, there are two panels for each heritage speaker group one in each language. The different filler types are indicated by different colors. As can be seen from the higher dark purple bars in Figure 6 a higher use of VN forms in female speech can be found in the speech of both mono- and bilingual speakers in the languages German and English. Figure 7 presents the VN ratios produced by the speaker groups in the three languages across two genders similar to Figure 6. The almost sole contribution of bilinguals to the VN ratio in Russian reported above raises the question of gender-related differences in this data set. There is no difference related to gender in the Russian monolingual data (female: n = 39, 6%; male: n = 13, 5%). The bilingual Russian speakers show distributional differences in VN filler forms in their Russian (Russian HSs in Germany female: *n* = 536, 39%; male: *n* = 256, 44%; Russian HSs in the U.S. female: *n* = 204, 23%; male: *n* = 114, 17%) yet to a lower degree than in their majority language (German of Russian HSs in Germany female: *n* = 577, 61%; male: *n* = 293, 43%; English of Russian HSs in the U.S. female: *n* = 276, 61%; male: *n* = 194, 43%).

**Figure 8:**
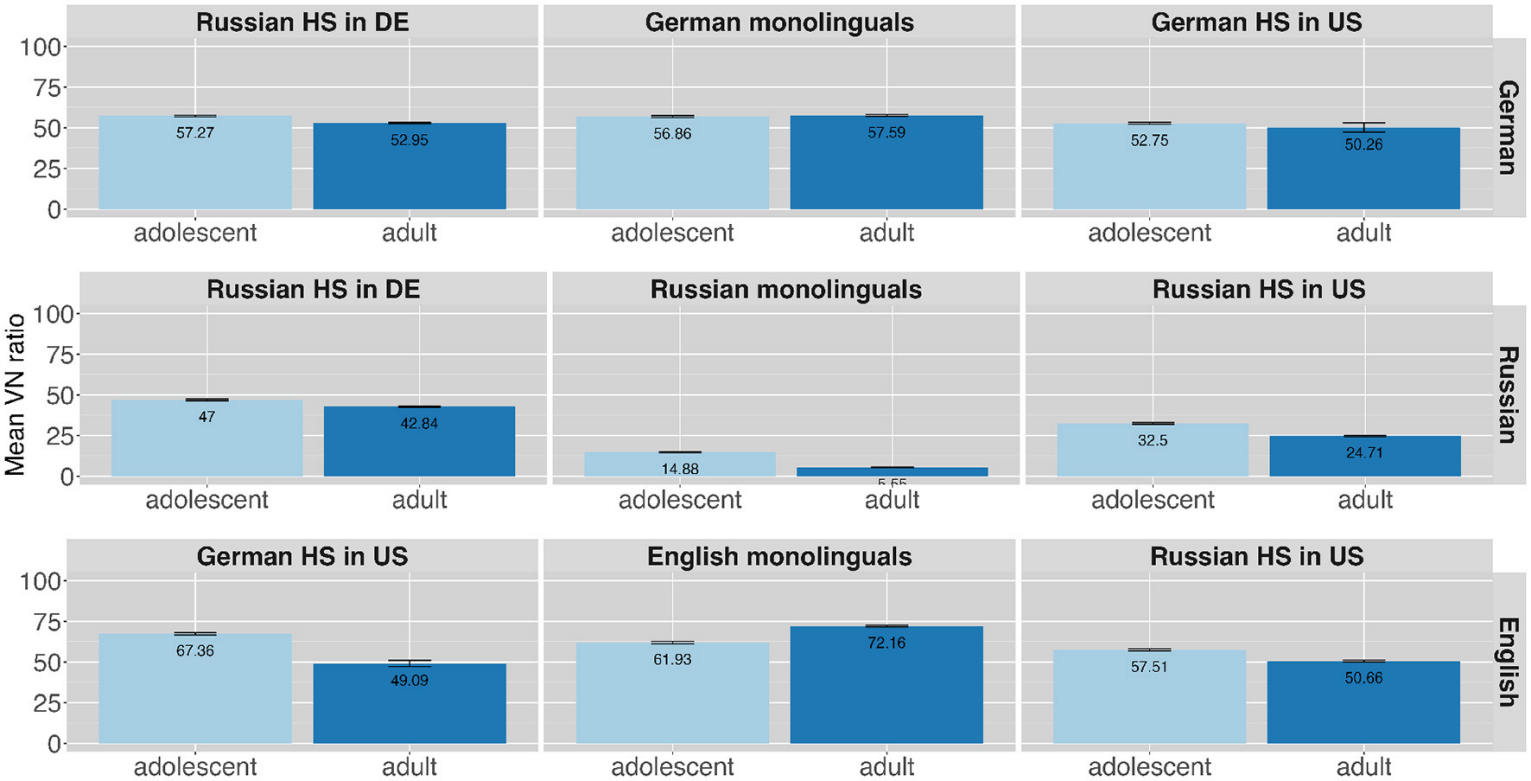
Distribution of VN variants across different speaker groups in the different languages (presented in rows) across two age groups.

Additionally, a difference in VN ratio can be observed comparing different age groups. While for the languages German (adolescents: *n* = 915, 56%; adults: *n* = 1,050, 55%) and English (adolescents: *n* = 608, 62%; adults: *n* = 648, 60%) this difference does not emerge, there is a difference in the Russian data (adolescents: *n* = 578, 31%; adults: *n* = 784, 25%). Figure 8 presents the VN ratio produced by the speaker groups in the three languages across two age groups. The plots are again arranged similar to Figure 6. Adolescents in Russian seem to produce more VN variants than adult speakers. The difference between the light and dark blue bars show that the difference across age groups in Russian is most pronounced in Russian monolingual speakers (adolescents: *n* = 57, 15%; adults: *n* = 28, 6%) compared to HSs in Germany (adolescents: *n* = 299, 47%; adults: *n* = 566, 43%) and the U.S. (adolescents: *n* = 222, 33%; adults: *n* = 190, 25%). A closer look at individual speaker groups also reveals a difference between age groups for the English of German HSs in the U.S. (adolescents: *n* = 210, 67%; adults: *n* = 98, 49%) Table 5 gives a more detailed overview of VN numbers and ratios for languages and speaker groups across two age groups.

A linear mixed regression was used to test if *bilingualism, language, gender* and *age group* as well as the random intercept of the *individual speaker* significantly predicted VN ratio. A model including random slopes for the speaker dependent variables *bilingualism, language, age group* and *gender* did not converge. We present the full model syntax of the model with the best fit which we used in the Supplementary Material. The model revealed significant main effects for *language* and *gender* as well as a significant interactions of *gender* and *language* as well as *bilingualism* and *language* and a three way interaction between *language, bilingualism* and *age group* (*p* < 0.001, conditional R^2^ = 0.55, marginal R^2^ = 0.33). A regression Table of the type III analysis of variance using the Satterthwaite's method is presented in Table 8.

**Table 8:** Type III analysis of variance table with Satterthwaite's method for VN ratio model.

	**Sum Sq**	**Mean Sq**	**NumDF**	**DenDF**	**F value**	**Pr(>F)**
Language	121993.51	60996.75	2.00	646.05	116.28	0.0000
Bilingual	1291.89	1291.89	1.00	344.86	2.46	0.1175
Gender	22267.18	22267.18	1.00	329.60	42.45	0.0000
Age_group	1575.89	1575.89	1.00	344.66	3.00	0.0839
Language:bilingual	31520.21	15760.10	2.00	624.36	30.04	0.0000
Language:gender	10083.48	5041.74	2.00	929.26	9.61	0.0001
Language:age_group	1014.52	507.26	2.00	628.60	0.97	0.3808
Bilingual:age_group	1471.87	1471.87	1.00	344.43	2.81	0.0948
Language:bilingual:age_group	3611.93	1805.96	2.00	625.36	3.44	0.0326

*Post-hoc* Tukey pairwise comparisons revealed a higher VN ratio for female speakers compared to male speakers in German and English respectively (German female: β = 21.7, SE = 3.8, df = 693.2, *t* = 5.6, *p* < 0.001; English female: β = 23.1, SE = 3.8, df = 692.9, *t* = 6.1, *p* < 0.001) as well as for female speakers of German and English compared to female speakers of Russian (German female: β = 36.5, SE = 3.0, df = 778.5, *t* = 12.1, *p* < 0.001; English female: β = 47.1, SE = 3.1, df = 793.6, *t* = 15.1, *p* < 0.001). The VN ratio is also higher for male speakers of German and English compared to male speakers of Russian (German male: β = 20.6, SE = 3.9, df = 892.9, *t* = 8.3, *p* < 0.001; English male: β = 29.8, SE = 3.7, df = 897.9, *t* = 7.9, *p* < 0.001). Additionally the VN ratio is lower for female speakers of Russian compared to male speakers of English and German (German male: β = −14.79, SE = 3.9, df = 536.9, *t* = −3.8, *p* < 0.001; English male: β = −24.0, SE = 3.8, df = 526.8, *t* = −6.3, *p* < 0.001) and for female German speakers compared to female English genders (German female: β = −10.7, SE = 3.3, df = 800.7, *t* = −3.2, *p* < 0.001).

For the three way interaction *bilingualism, language* and *age group* the *post hoc* Tukey test revealed higher VN ratios for monolingual speakers of German and English compared to monolingual Russian speakers for both age groups respectively (monolingual German adolescents: β = 40.8, SE = 6.8, df = 222.7, t = 6.0, *p* < 0.001; monolingual German adults: β = 49.4, SE = 5.7, df = 226.4, *t* = 8.7, *p* < 0.001; monolingual English adolescents: β = 45.7, SE = 7.1, df = 227.3, *t* = 6.4, *p* < 0.001, monolingual English adults: β = 66.0, SE = 5.7, df = 230.0, *t* = 11.6, *p* < 0.001) as well as compared to bilingual Russian speakers (monolingual German adolescents: β = 22.4, SE = 5.6, df = 349.1, *t* = 4.0, *p* < 0.001; monolingual German adults: β = 21.3, SE = 4.9, df = 230.3, *t* = 4.3, *p* < 0.001; monolingual English adolescents: β = 20.6, SE = 6.1, df = 248.8, *t* = 3.3, *p* < 0.001, monolingual English adults: β = 37.9, SE = 4.9, df = 235.4, *t* = 7.7, *p* < 0.001). Additionally VN ratios are higher for bilingual speakers of German and English compared to bilingual speakers of Russian for both age groups respectively (bilingual German adolescents: β = 18.3, SE = 4.4, df = 571.9, *t* = 4.2, *p* < 0.001; bilingual German adults: β = 12.5, SE = 3.8, df = 769.9, *t* = 3.3, *p* < 0.001; bilingual English adolescents: β = 24.2, SE = 3.5, df = 723.8, *t* = 7.0, *p* < 0.001, bilingual English adults: β = 17.9, SE = 3.7, df = 740.9, *t* = 4.9, *p* < 0.001). Compared to monolingual Russian the VN ratio is lower for bilingual speakers of German, English and Russian also for both age groups respectively (bilingual German adolescents: β = −36.6, SE = 5.9, df = 259.9, *t* = −6.2, *p* < 0.001; bilingual German adults: β = −40.5, SE = 5.3, df = 296.9, *t* = −7.6, *p* < 0.001; bilingual English adolescents: β = −49.4, SE = 5.9, df = 259.2, *t* = −8.4, *p* < 0.001, bilingual English adults: β = −45.9, SE = 5.3, df = 293.1, *t* = −8.7, *p* < 0.001; bilingual Russian adolescents: β = −25.1, SE = 5.8, df = 249.9, *t* = −4.3, *p* < 0.001, bilingual Russian adults: β = −28.1, SE = 4.8, df = 229.4, *t* = −5.8, *p* < 0.001)

The data analysis reveals VN ratio as a language-specific aspect of filler particles modulated by gender as well as being related to bilingualism and age group. The VN filler variant shows higher use in German and English with low rates for Russian. Overall, the VN ratio is higher for female speakers in German and English, while there does not seem to be a difference related to gender in the Russian data. However, an age group difference emerged in Russian monolingual speakers. Heritage speakers of Russian show higher uses of the VN variant in their HL even if lower compared to the ML. Bilingual heritage speakers produce similar gender-related distributional patterns compared to monolinguals in both their languages, showing language-specific use of FP form related to gender.

## 4 Discussion

The current study investigated the frequency and form of FPs in different languages and language contact situations. We found in general a higher FP ratio in the Russian data and in bilingual and formal speech within the data considered from the RUEG corpus. Additionally, we found higher VN ratios in the German and English compared to Russian data as well as higher VN ratios in female compared to male speech, though this effect was restricted to German and English language use.

Addressing research question 1, we found higher FP frequencies in the narrations produced by bilingual heritage speakers compared to monolingual speakers in the respective language, confirming H1a. Additionally, we found higher FP frequency in the HL compared to the ML of the bilingual speakers. This could be related to the language dominance as a result of societal status of the bilinguals' languages. The minority HL for which there are restricted opportunities, i.e., less contexts and people in everyday life, to use might pose more speech planning effort compared to the more frequently and wider used ML. The more frequent use of FPs could also be related to higher cognitive effort monitoring two languages in general as suggested by Kroll and Gollan (2014). The interpretation of FP frequency as an indicator of fluency as a result of higher cognitive load is in line with the observation of higher FP ratio in bilingual speech in other studies (e.g., (Gilquin, 2008; de Jong, 2016, 2018). Whether the higher use in one of the speakers' languages is noticable to listeners would need to be investigated in a perception study. This could provide further evidence for aspects contributing to a perceived non-native accent (Kupisch et al., 2014).

The results also showed higher FP frequencies unrelated to language background and bilingualism. Higher FP use was found in formal narrations and monolinguals show a similar increase of FPs which could be explained by cognitive load. Our H1b was therefore also supported.

In the formal contexts speakers are assumed to be more careful regarding speech planning; they probably try to be as precise as possible, and they may also plan their productions carefully in regards to speech style and register. Monolingual majority speakers as well as bilingual heritage speakers are under pressure to find the right words. In similar contexts of high demands on lexical retrieval other groups of mono- and bilinguals also behaved similarly with regard to FP frequency (de Jong, 2016). While the elicitation situation did not include a real police officer, it is possible that speakers were less at ease in these formal situations than in an informal setting as well. This increase of interpersonal uncertainty may also lead to an increase in filler frequency (Rochester, 1973). FP use may, therefore, reflect a person's stress level, as suggested by Vasilescu and Adda-Decker (2006), which can be influenced by the formality of a situation, the cognitive load of speech planning and language monitoring.

Our results contrast with some prior findings about the higher use of FPs in informal compared to formal register (Crible et al., 2017). It is important to note that formal register in prior studies like Crible et al. (2017) investigated prepared speech like political speeches. While this is a formal spoken register, it is usually based on written scripts. Conceptual speech planning in these cases, therefore, is settled prior to speech production, resulting in lower cognitive load and possibly lower level of stress. Further research on speech planning and production, therefore, will need to take into account different ways of defining formality and also different factors influencing cognitive load and stress level [see Defrancq and Plevoets (2017) for a discussion on cognitive load and fillers in language interpreters].

The higher use of FPs in formal situations was also found in the Turkish data of the RUEG corpus (Özsoy and Blum, 2023). This study found discourse markers and FPs to be more frequent in formal compared to informal narrations. They explain the higher frequency in these cases with macro planning efforts which might be higher in formal situations i.e. when giving an accurate police report compared to informal speech addressing a friend as is the case in the RUEG elicitation method. This is in line with our findings of higher FP frequency in formal narrations for the three different languages Russian, English and German. Additionally, Özsoy and Blum (2023) found higher use of discourse markers and fillers in utterance initial position. In utterance-initial position, FPs are assumed to signal macro planning pauses, or major delays in speech production (Clark and FoxTree, 2002). In this position and function, prior work found VN fillers to be used more frequently (Clark and FoxTree, 2002; Kjellmer, 2003). This would suggest a higher use of VN fillers in formal narrations. Our analysis of filler form, however, did not focus on variation related to formality but rather on language-specific filler choice and socio-linguistic marking of gender as relevant factors for filler form. Further analysis of the fillers' form and their distribution in utterance initial vs. internal position in our data would be necessary to draw further conclusions. The analysis of utterance initial vs. utterance internal FP use would also shed more light on the FP frequency difference between speaker groups. Prior work by de Jong (2016) found FP frequency differences only utterance internal while utterance initially the frequency of FPs did not differ between mono- and bilingual speakers. While this was not the focus of this investigation, a first analysis of the same corpus data suggests similar tendencies for these heritage bilingual speakers.

Recent research on the parallel between FPs and lexicalized discourse markers (e.g., *well, so, like, yeah* in English) along with their functional aspects in spontaneous discourse has also been carried out using data from the RUEG corpus. First investigations of discourse boundaries suggest they might be in complementary distribution across different registers (Labrenz et al., 2023). This aspect might further influence the higher number of FPs in formal narrations. A more detailed analysis of speech context would be necessary to determine which of the FPs in our data are proper speech planning hesitations and which ones are used to structure and organize discourse.

Higher FP use was also found in narrations by older speakers within our data set. While there was no such difference in the monolingual speech, bilingual adults produced more FPs than bilingual adolescents. This age related effect could also be connected to cognitive load. Previous research has shown an increase of speech disfluency in the speech of older compared to younger speakers (see Mortensen et al., 2006 for an overview). Our results are in line with these, however, the age groups in our data differ in age ranges to prior work: the older age group in our analysis matches the young adult group in some prior work (Bortfeld et al., 2001). Since the age group differences only emerge in the HS group another explanation could be their language use. Adolescents might still be living with their HL speaking parents while attending a ML dominant educational context. The younger speakers might therefore be more balanced bilinguals and more used to the language use in both their languages. Adult HSs on the other hand might no longer live with their parents, i.e. less immersed in both their languages resulting in less habitual ease and possibly higher speech planning effort in this group of bilinguals. Further analysis of the speakers' language use pattern would be necessary too draw further conclusions. The difference between age groups observed in our data could also be related to speech style within these groups. As such the use of more FPs would be an indicator of a social group, in this case age. The results of the FP segmental form provide further evidence for this interpretation.

In line with a filler-as-symptom approach, the increased use of FPs in formal narrations and older bilingual speech can be interpreted related to cognitive effort and speech macro planning. However, both results can also be linked to a filler-as-signal interpretation: the use of FPs informal narrations can be interpreted as a feature of a speech register, and of a speech style of a specific age and social group. Whether or not the increased use of FPs is linked to one or the other, especially the differences across different formality levels is present in the speech of mono- and bilingual speakers of the three languages investigated here. This suggests more similarities than differences in the use of FPs between the speaker groups in our data, in line with other work on heritage speakers and register (Wiese et al., 2022; Özsoy and Blum, 2023).

Our second research question was concerned with the language-specificity of filler forms. As predicted, the data presented a language-specific preference. There were language-specific VN ratios and a clear distinction of low VN use in Russian and higher VN use in the Germanic languages German and English. So H2a could be confirmed based on our analysis. This is in line with earlier reports on language-specific higher VN ratios for English and German compared to, e.g., a V preference in Dutch (de Leeuw, 2007) and French (Torreira et al., 2010). Our analysis adds to this an observed V preference in Russian. The current study did not include acoustic details; further research on the vowel formants could provide more insight into the language-specific fillers and potentially reveal differences between the English and German fillers and between fillers in the two languages of the bilingual heritage speakers in the RUEG corpus.

While the monolingual Russian speakers show a V preference in filler production, the bilingual heritage speakers of Russian in our data do produce the VN form in their heritage language which is an unusual FP form for Russian. This can be interpreted as a form of transfer from the majority languages German and English. However, they do show a sensitivity for the language-specific FP preference. Heritage speakers of Russian produce fewer VN fillers in their Russian compared to their English, showing an understanding of language-specific usage patterns. Thus H2c was also partly confirmed: heritage speakers show language-specific preference for the V form yet not a V ratio similar to monolingual speakers. This result is consistent with prior research on bilinguals' FP use with lower VN ratio in the L2 in cases of V preference in the L1, e.g., in French learners of English (Gilquin, 2008), French-German bilinguals (Lo, 2020) and Spanish-English bilinguals (Muhlack, 2023).

The transfer of a relatively non-salient feature like a filled pause from the majority to the heritage language could be one of the contributing factors to a perceived heritage accent (Kupisch et al., 2014). While heritage speakers are said to show native-like segmental features in their heritage language, they are easily detected by monolingual listeners. Further perception studies on the perceived accent of HL speakers related to the use of fillers could provide further insights in this area of heritage speakers and heritage languages.

The investigation of VN ratio additionally revealed patterns related to the socio-linguistic parameters and confirmed H2b. The variable age group was included in our analysis, yet, the results are not very conclusive. For aspect of gender, however, the analysis did reveal gender and language related effects. In the Germanic languages English and German female speakers produce more VN than V forms while male speakers produce VN and V forms in equal distribution in these two languages. This higher use of VN variants in female speech has previously been reported for both languages (Acton, 2011; Tottie, 2011; Fruehwald, 2016; Wieling et al., 2016; Belz, 2021). The data analyzed here confirms this, along with a previously reported higher FP frequency overall in male compared to female speakers Wieling et al. (2016). Our analysis suggests that the lexical form of FPs can be considered a socio-linguistic marker of gender in these languages (Fruehwald, 2016). The same difference across gender was not found in the Russian data. For monolingual Russian, FP variants can therefore not be considered socio-linguistic markers of gender based on the analyzed data. This socio-linguistic difference between their heritage and their majority language is acquired by heritage speakers of Russian, both in the United States and Germany. While heritage speakers produce higher ratios of VN fillers in their Russian, showing a lexical transfer of the items themselves, they do not appear to transfer the gender-specific preferences from their ML to their heritage Russian or vice versa. The heritage speakers analyzed here, therefore, show an awareness of this socio-linguistic phenomenon in both their languages even though fillers are not very salient or easily detected in speech.

In our analysis we focus on groups and group variables and include individual speakers as a random effect. The use of FPs has, however, also been shown to be idiosyncratic (Braun et al., 2023; Özsoy and Blum, 2023). In an analysis Braun et al. (2023) used disfluency parameters among others also FP use in a forensic approach to successfully identify their 8 female speakers. A closer look at the individual FP usage strategies of the speakers within the RUEG corpus could provide further insight into this area and application of inter-speaker variability. Especially to see whether speakers tend to share idiosyncratic FP use across their two languages based on the language specific FP use which emerged in our group analysis.

This study adds to the growing body of research on heritage speaker grammar, and more specifically to the area of discourse pragmatics and speech planning. While many studies have looked at heritage speakers' productions in their ML and HL (Hlavac, 2011; Lo, 2020) or only considered one of the bilinguals' languages (Pinto and Raschio, 2007), the current study includes different language pairs and also includes monolingual speakers as comparison group. The elicitation method with different formalities is shared across these speaker groups which enables an investigation of variation among monolingual speakers of different languages. For the area of fillers the results presented here are in line with the native speaker continuum (Wiese et al., 2022). Heritage speakers do show language-specific filler pattern usage even if they deviate from the monolinguals living in a different country. This is not surprising since the language input and the linguistic peer group also differ. At the same time, the results suggest language transfer, especially of the VN form. Both results support the word status of FPs in different languages in the sense that they need to and can be acquired in a language. Further analysis should take into account the different discourse functions of the FPs analyzed here. This could provide insight into the semantics and pragmatics of these items, and whether the range differs between heritage and monolingual speakers. Research on lexicalized discourse markers suggests that different functional ranges are also related to different degrees of formality (Labrenz et al., 2021). One function of FPs supported in this study is the use of different filler forms as a socio-linguistic marker related to gender. The bilingual speakers in the corpus data analyzed here acquire this pragmatic function. Therefore, it is possible that other discourse pragmatic functions are also acquired by heritage speakers.

## 5 Conclusion and outlook

This study addresses the use of fillers in majority and heritage language use. Three observations can be drawn from the analysis: filler particle frequency is related not only to bilingualism but also to formality of the situation. This factor influencing filler particle frequency can be related to cognitive load and is in line with the filler-as-symptom approach. It is also compatible with prior work on bilinguals, and highlights the fact that cognitive load rather than language proficiency are at play when filler frequency is increased, since monolingual speakers also show higher filler frequencies when speaking in a formal setting. An alternative explanation, in line with the filler-as-signal view, is that filler particle frequency reflects aspects of speech style or register related to formal situations. The filler particle form was observed to be language-specific in terms of the preference for a vocalic or a vocalic-nasal variant. The latter is the preferred form in English and German while the former is the predominant form in Russian. Heritage speakers seem to be aware of language-specific tendencies but transfer an increased use of vocalic-nasal forms from the majority language to their heritage Russian. Additionally, the differences in filler particle form across gender suggests that this serves as a socio-linguistic marker in English and German, but not in Russian. This language-specific socio-linguistic difference is acquired and produced by heritage speakers of Russian when they speak the majority language. Future work will include a closer look at the vowel qualities of the fillers in the three languages, investigating whether heritage speakers not only share the segmental structure of fillers with monolingual speakers but addressing the language-specific filler forms in more detail.

## Data availability statement

Publicly available datasets were analyzed in this study. This data can be found here: RUEG corpus downloadable via Zenodo (doi: 10.5281/zenodo.3236068) and openly accessible via the browser-based application ANNIS (https://korpling.german.hu-berlin.de/annis/#c=rueg). The data analyzed in this study comprises query results performed on the subcorpora RUEG-EN 1.0- SNAPSHOT, RUEG-DE 1.0-SNAPSHOT, and RUEG-RU 1.0-SNAPSHOT.

## Ethics statement

Ethical review and approval was not required for the study on human participants in accordance with the local legislation and institutional requirements. Written informed consent from the patients/ participants or patients/participants' legal guardian/next of kin was not required to participate in this study in accordance with the national legislation and the institutional requirements.

## Author contributions

MB: Writing – original draft, Methodology, Formal analysis. MZ: Supervision, Writing – review & editing, Methodology.

## Footnotes

^1^English sub-corpus: https://korpling.org/annis/#_c=UlVFRy1FTl8xLjAtU05BUFNIT1Q; German sub-corpus: https://korpling.org/annis/#_c=UlVFRy1ERV8xLjAtU05BUFNIT1Q; Russian sub-corpus: https://korpling.org/annis/#_c=UlVFRy1SVV8xLjAtU05BUFNIT1Q.

^2^There were only 3 data points of speakers of non-binary gender. The data analysis therefore shows only two genders, with no claim of completeness regarding the representation of genders.

## References

[B1] Acton E. K. (2011). “On gender differences in the distribution of um and uh,” in University of Pennsylvania Working Papers in Linguistics: Selected Papers From NWAV, Vol. 17 (Pennsylvania: University of Pennsylvania).

[B2] Bates D. Mächler M. Bolker B. Walker S. (2015). Fitting linear mixed-effects models using lme4. J. Stat. Softw. 67, 1–48. 10.18637/jss.v067.i01

[B3] Belz M. (2021). “Die phonetik von äh und ähm,” in Akustische Variation von Füllpartikeln im Deutschen (Berlin; Heidelberg: J.B. Metzler). 10.1007/978-3-662-62812-6

[B4] Belz M. (2023). Defining filler particles: a phonetic account of the terminology, form, and grammatical classification of “filled pauses. Languages 8, 57. 10.3390/languages8010057

[B5] Belz M. Odebrecht C. (2022). Abschnittsweise analyse sprachlicher Flüssigkeit in der Lernersprache: Das Ganze ist weniger informativ als seine Teile. Zeitschrift für germanistische Linguistik 50, 131–158. 10.1515/zgl-2022-2051

[B6] Belz M. Reichel U. (2015). “Pitch characteristics of filled pauses,” in The 7th Workshop on Disfluency in Spontaneous Speech.

[B7] Berthold A. Jameson A. (1999). “Interpreting symptoms of cognitive load in speech input,” in UM99 User Modeling. CISM International Centre for Mechanical Sciences, ed J. Kay (Vienna: Springer), 235–244.

[B8] Betz S. Bryhadyr N. Türk O. Wagner P. (2023). Cognitive load increases spoken and gestural hesitation frequency. Languages 8, 71. 10.3390/languages8010071

[B9] Bialystok E. (2017). The bilingual adaptation: how minds accommodate experience. Psychol. Bull. 143, 233–262. 10.1037/bul0000099 28230411 PMC5324728

[B10] Bortfeld H. Leon S. D. Bloom J. E. Schober M. F. Brennan S. E. (2001). Disfluency rates in conversation: effects of age, relationship, topic, role, and gender. Lang. Speech 44, 123–147. 10.1177/00238309010440020101 11575901

[B11] Braun A. Elsässer N. Willems L. (2023). Disfluencies revisited are they speaker-specific? Languages 8, 155. 10.3390/languages8030155

[B12] Candea M. Vasilescu I. Adda-Decker M. (2005). “Inter- and intra-language acoustic analysis of autonomous fillers,” in Proceedings From the Disfluency in Spontaneous Speech DiSS 2005 (Aix-en-Provence: Equipe DELIC Université de Provence), 47–52.

[B13] Clark H. H. FoxTree J. E. (2002). Using uh and um in spontaneous speaking. Cognition 84, 73–111. 10.1016/S0010-0277(02)00017-3 12062148

[B14] Corley M. Stewart O. W. (2008). Hesitation disfluencies in spontaneous speech: the meaning of um. Lang. Linguist. Comp. 2(4), 589–602. 10.1111/j.1749-818X.2008.00068.x

[B15] Crible L. Degand L. Gilquin G. (2017). The clustering of discourse markers and filled pauses. Lang. Contrast 17, 69–95. 10.1075/lic.17.1.04cri

[B16] de Boer M. M. Heeren W. F. L. (2020). Cross-linguistic filled pause realization: the acoustics of ”uh” and ”um” in native Dutch and non-native English. J. Acoust. Soc. Am. 148, 3612–3622. 10.1121/10.0002871 33379906

[B17] de Jong N. H. (2016). Predicting pauses in L1 and L2 speech: the effects of utterance boundaries and word frequency. Int. Rev. Appl. Linguist. Lang. Teach. 54, 2. 10.1515/iral-2016-9993

[B18] de Jong N. H. (2018). Fluency in second language testing: insights from different disciplines. Lang. Assess. Q. 15, 237–254. 10.1080/15434303.2018.1477780

[B19] de Jong N. H. Groenhout R. Schoonen R. Hulstijn J. H. (2013). Second language fluency: Speaking style or proficiency? Correcting measures of second language fluency for first language behavior. Appl. Psycholinguist. 36, 223–243. 10.1017/S0142716413000210

[B20] de Leeuw E. (2007). Hesitation Markers in English, German, and Dutch. J. Germanic Linguist. 19, 85–114. 10.1017/S1470542707000049

[B21] Defrancq B. Plevoets K. (2017). “Over-uh-load, filled pauses in compounds as a signal of cognitive load,” in Making Way in Corpus-based Interpreting Studies, eds. M. Russo, and C. Bendazzoli (Singapore: Springer), 43–64.

[B22] Fischer K. (2000). Discourse particles, turn-taking, and the semantics-pragmatics interface. Rev. Sémant. Pragmat. 8, 111–132.

[B23] Fischer K. Niebuhr O. Novák-Tót E. Jensen L. C. (2017). “Strahlt die negative Reputation von Häsitationsmarkern auf ihre Sprecher aus?,” in Proceedings of the 43rd Annual Conference of The German Acoustical Society (Kiel: DAGA), 1450–1453.

[B24] FoxTree J. E. (2001). Listeners' uses of um and uh in speech comprehension. Memory Cognit. 29, 320–326. 10.3758/BF03194926 11352215

[B25] FoxTree J. E. (2002). Interpreting pauses and ums at turn exchanges. Discour. Proc. 34, 37–55. 10.1207/S15326950DP3401_2

[B26] Fraundorf S. H. Watson D. G. (2011). The disfluent discourse: effects of filled pauses on recall. J. Mem. Lang. 65, 161–175. 10.1016/j.jml.2011.03.004 21765590 PMC3134332

[B27] Fruehwald J. (2016). “Filled pause choice as a sociolinguistic variable,” in University of Pennsylvania Working Papers in Linguistics: Selected Papers from NWAV, Vol.22 (Pennsylvania: University of Pennsylvania), 6.

[B28] Gilquin G. (2008). Hesitation markers among EFL learners: pragmatic deficiency or difference? Pragmat. Corpus Linguist: A Mutualistic Entente. 2, 119–149. 10.1515/9783110199024.119

[B29] Hlavac J. (2011). Hesitation and monitoring phenomena in bilingual speech: a consequence of code-switching or a strategy to facilitate its incorporation? J. Pragmat. 43, 3793–3806. 10.1016/j.pragma.2011.09.008

[B30] Kjellmer G. (2003). Hesitation, in defence of ER and ERM. Engl. Stud. 84, 170–198. 10.1076/enst.84.2.170.14903

[B31] Knudsen B. Creemers A. Meyer A. S. (2020). Forgotten little words: how backchannels and particles may facilitate speech planning in conversation? Front. Psychol. 11:593671. 10.3389/fpsyg.2020.593671 33240183 PMC7677452

[B32] Korenar M. Treffers-Daller J. Pliatsikas C. (2023). Two languages in one mind: Insights into cognitive effects of bilingualism from usage-based approaches. Nase Rec 106, 1. 10.58756/n11062303 PMC9974958 36854883

[B33] Krause T. Zeldes A. (2016). ANNIS3: a new architecture for generic corpus query and visualization. Digit. Scholarsh. Hum. 31, 118–139. Available online at: http://dsh.oxfordjournals.org/content/31/1/118

[B34] Kroll J. F. Gollan T. H. (2014). “Speech planning in two languages: what bilinguals tell us about language production,” in The Oxford Handbook of Language Production, eds. M. Goldrick, V. S. Ferreira (Oxford: Oxford University Press), 165–181.

[B35] Kupisch T. Barton D. Hailer K. Klaschik E. Stangen I. Lein T.. (2014). Foreign accent in adult simultaneous bilinguals. Heritage Lang. J. 11, 123–150. 10.46538/hlj.11.2.2

[B36] Kuznetsova A. Brockhoff P. B. Christensen R. H. B. (2017). lmerTest package: tests in linear mixed effects models. J. Stat. Softw. 82, 1–26. 10.18637/jss.v082.i13

[B37] Labrenz A. Böttcher M. Groth F. Iefremenko K. Katsika K. (2023). “Discourse markers and filler particles at the boundary: a corpus study across languages, speaker groups, and communicative situations,” in Discourse Markers - Theories and Methods (Paris).

[B38] Labrenz A. Katsika K. Iefremenko K. Wiese H. Schroeder C. Allen S. (2021). “Functional variation and change of discourse-pragmatic markers in heritage speakers' two languages “a comparative corpus study,” in Discourse-Pragmatic Variation & *Change (DiPVaC)* (Melbourne, VIC).

[B39] Lenth R. V. (2022). “Emmeans: estimated marginal means, aka least-squares means,” in R Package Version 1.7.4–1.

[B40] Levelt W. J. M. (1983). Monitoring and self-repair in speech. Cognition 14, 41–104. 10.1016/0010-0277(83)90026-4 6685011

[B41] Lickley R. J. (2015). “Fluency and disfluency,” in The Handbook of Speech Production, eds M. A. Redford (Oxford: John Wiley & Sons, Ltd.), 445–474.

[B42] Lo J. J. H. (2020). Between “Äh(m)” and “Euh(m)”: the distribution and realization of filled pauses in the speech of german-french simultaneous bilinguals. Lang. Speech 63, 746–768. 10.1177/0023830919890068 31789576

[B43] Maclay H. Osgood C. E. (1959). Hesitation phenomena in spontaneous English speech. Word 15, 19–44. 10.1080/00437956.1959.11659682

[B44] Montrul S. (2015). The Acquisition of Heritage Languages. Cambridge: Cambridge University Press.

[B45] Mortensen L. Meyer A. S. Humphreys G. W. (2006). Age-related effects on speech production: a review. Lang. Cogn. Process. 21, 238–290. 10.1080/01690960444000278

[B46] Muhlack B. (2023). “Filler particles in English and Spanish L1 and L2 speech,” in Proceedings of ICPhS 2023, eds R. Skarnitzl and J. Volín (Prague: GUARANT International spol. s r.o.), 24182422.

[B47] Muysken P. (2013). Language contact outcomes as the result of bilingual optimization strategies. Biling.: Lang. Cogn. 16, 709–730. 10.1017/S1366728912000727

[B48] Navarretta C. (2015). “The function of fillers, filled pauses and co-occurring gestures in danish dyadic conversations,” in Proceedings from the 3rd European Symposium on Multimodal Communication, Dublin, September 2015, eds E. Gilmartin, L. Cerrato, and N. Campbell (Linköping: Linköping Electronic Conference Proceedings), 55–61.

[B49] Niebuhr O. Fischer K. (2019). “Do not hesitate! —unless you do it shortly or nasally: how the phonetics of filled pauses determine their subjective frequency and perceived speaker performance,” in Interspeech 2019 (North Bethesda: ISCA), 544–548.

[B50] Özsoy O. Blum F. (2023). Exploring individual variation in Turkish heritage speakers' complex linguistic productions: Evidence from discourse markers. Appl. Psycholinguist. 2023, 1–31. 10.1017/S0142716423000267

[B51] Pinto D. Raschio R. (2007). A comparative study of requestsin heritage speaker Spanish, L1 Spanish,and L1 English. Int. J. Bilingual. 11, 135–155. 10.1177/13670069070110020101

[B52] Pistor T. (2016). Prosodic Universals in Discourse Particles. Boston: Speech Prosody, 869-873.

[B53] Polinsky M. (2018). Heritage Languages and Their Speakers. Cambridge: Cambridge University Press.

[B54] Posit team (2023). RStudio: Integrated Development Environment for R. Boston: Posit Software, PBC.

[B55] R Core Team (2023). R: A Language and Environment for Statistical Computing. Vienna: R Foundation for Statistical Computing.

[B56] Rochester S. R. (1973). The significance of pauses in spontaneous speech. J. Psycholinguist. Res. 2, 51–81. 10.1007/BF01067111 24197795

[B57] Rose R. Watanabe M. (2019). “A crosslinguistic corpus study of silent and filled pauses: when do speakers use filled pauses to fill pauses?,” in Proceedings of ICPhS 2019, eds S. Calhoun, P. Escudero, M. Tabain, and P. Warren (Canberra, ACT: Australasian Speech Science and Technology Association Inc.), 2615–2619.

[B58] Schegloff E. A. (2010). Some other “uh (m)” s. Discourse Proc. 47, 130–174. 10.1080/01638530903223380

[B59] Shriberg E. (2001). To ‘errrr' is human: ecology and acoustics of speech disfluencies. J. Int. Phon. Assoc. 31, 153–169. 10.1017/S0025100301001128

[B60] Sorace A. (2011). Pinning down the concept of “interface” in bilingualism. Linguist. Approach. Bilingual. 1, 1–33. 10.1075/lab.1.1.01sor

[B61] Staley L. Jucker A. H. (2021). “The uh deconstructed pumpkin pie”: the use of uh and um in Los Angeles restaurant server talk. J. Pragmat. 172, 21–34. 10.1016/j.pragma.2020.11.004

[B62] Stepanova S. (2007). “Some features of filled hesitation pauses in spontaneous Russian,” in Proceedings of the 16th International Congress of Phonetic Sciences, Saarbrücken, 2007, eds J. Trouvain and W. J. Barry, 1325–1328. Available online at: www.icphs2007.de

[B63] Swerts M. (1998). Filled pauses as markers of discourse structure. J. Pragmat. 30, 485–496. 10.1016/S0378-2166(98)00014-9

[B64] Torreira F. Adda-Decker M. Ernestus M. (2010). The Nijmegen corpus of casual French. Speech Commun. 52, 201–212. 10.1016/j.specom.2009.10.004

[B65] Tottie G. (2011). Uh and Um as sociolinguistic markersin British English. Int. J. Corpus Linguist. 16, 173–197. 10.1075/ijcl.16.2.02tot

[B66] Tottie G. (2014). On the use of uh and um in American English. Funct. Lang. 21, 6–29. 10.1075/fol.21.1.02tot

[B67] van Rijswijk R. Muntendam A. Dijkstra T. (2017). Focus marking in Dutch by heritage speakers of Turkishand Dutch L1 speakers. Journal ofPhonetics 61:48–70. 10.1016/j.wocn.2017.01.003

[B68] Vasilescu I. Adda-Decker M. (2006). “Language, gender, speaking style and language proficiency as factors influencing the autonomous vocalic filler production in spontaneous speech,” in Interspeech 2006 (North Bethesda: ISCA), 1850–1853.

[B69] Vasilescu I. Nemoto R. Adda-Decker M. (2007). “Vocalic hesitations vs. vocalic systems: a cross-language comparison,” in Proceedings of the 16th International Congress of Phonetic Sciences, Saarbrücken, 2007, eds J. Trouvain and W. J. Barry, 1101–1104. Available online at: www.icphs2007.de

[B70] Watanabe M. Den Y. Hirose K. Minematsu N. (2005). “The effects of filled pauses on native and non-native listeners' speech processing,” in Proceedings of DiSS'05, Disfluency in Spontaneous Speech Workshop, 169–172.

[B71] Wickham H. (2016). ggplot2: Elegant Graphics for Data Analysis. New York: Springer-Verlag.

[B72] Wieling M. Grieve J. Bouma G. Fruehwald J. Coleman J. Liberman M. (2016). Variation and change in the use of hesitation markers in germanic languages. Lang. Dynam. Change 6, 199–234. 10.1163/22105832-00602001

[B73] Wiese H. (2020). “Language situations: a method for capturing variation within speakers' repertoires,” in Methods in Dialectology XVI, ed. Y. Asahi (Frankfurt: Peter Lang), 105–121.

[B74] Wiese H. Alexiadou A. Allen S. Bunk O. Gagarina N. Iefremenko K.. (2021). RUEG Corpus. 10.3389/fpsyg.2021.717973 PMC8865415 35222135

[B75] Wiese H. Alexiadou A. Allen S. Bunk O. Gagarina N. Iefremenko K.. (2022). Heritage speakers as part of the native language continuum. Front. Psychol. 12, 717973. 10.3389/fpsyg.2021.717973 35222135 PMC8865415

