# Supplementary material for: Do you say uh or uhm? A cross-linguistic approach to filler particle use in heritage and majority speakers across three languages
Source: Front Psychol. 2024 Mar 11;15:1305862. doi: 10.3389/fpsyg.2024.1305862 (PMC10986790; doi:10.3389/fpsyg.2024.1305862)
Supplement: Supplementary file 1 [file Data_Sheet_1.pdf]

frontiers

Supplementary Material

1 SUPPLEMENTARY CODE

1.1 Queries in the RUEG Corpus

The following queries were used for the filler particle occurrence, frequency and word frequency (see 1., 2. and 3. respectively for each sub corpus) and were performed via the browser-based application ANNIS `https://korpling.german.hu-berlin.de/annis/#c=rueg`:

For the English data RUEG-EN_1.0-SNAPSHOT:

1. *dipl* = /(ah|ah:|a:h|a:h:|eh|e:h|e:h:|er|er:|e:r|e:r:|
   uh|u:h|uh:|u:h:|um|uhm|u:m|um:|uhm:|u:hm|u:m:|
   uh:m:|em|e:m|em:|e:m:|mm|mm:)/
2. *dipl* = /(ah|ah:|a:h|a:h:|eh|e:h|e:h:|er|er:|e:r|e:r:|
   uh|u:h|uh:|u:h:|um|uhm|u:m|um:|uhm:|u:hm|u:m:|
   uh:m:|em|e:m|em:|e:m:|mm|mm:)/ @* *doc*
3. *norm*! = "/(\.|, |; |?|!)/" _ = _
   dipl! = /(ah|ah:|a:h|a:h:|eh|e:h|e:h:|er|er:|e:r|e:r:|
   uh|u:h|uh:|u:h:|um|uhm|u:m|um:|uhm:|u:hm|u:m:|
   uh:m:|em|e:m|em:|e:m:|mm|mm:)/ @* *doc*

For the German data RUEG-DE_1.0-SNAPSHOT:

1. *dipl* = /(äh|ä:h|äh:|ä:h:|ähm|ä:hm|ähm:|ä:hm:|
   öh|ö:h|öhm|ö:hm|ö:hm:|hm|hm:|mh|m:h)/
2. *dipl* = /(äh|ä:h|äh:|ä:h:|ähm|ä:hm|ähm:|ä:hm:|
   öh|ö:h|öhm|ö:hm|ö:hm:|hm|hm:|mh|m:h)/ @* *doc*
3. *norm*! = "/(\.|, |; |?|!)/" _ = _
   dipl! = /(äh|ä:h|äh:|ä:h:|ähm|ä:hm|ähm:|ä:hm:|
   öh|ö:h|öhm|ö:hm|ö:hm:|hm|hm:|mh|m:h)/ @* *doc*

For the Russian data RUEG-RU_1.0-SNAPSHOT:

1. *dipl* = /(эм|эм:|э:м:|э:м|хм|х:м|хм:|х:м:|
   э:|а:|а|ам|а:м|ам:|а:м:|м|м:)/
2. *dipl* = /(эм|эм:|э:м:|э:м|хм|х:м|хм:|х:м:|
   э:|а:|а|ам|а:м|ам:|а:м:|м|м:)/ !_ = _ *norm* = /.*/? & *doc* & #1 @* #3
3. *norm*! = "/(\.|, |; |?|!)/" _ = _
   dipl! = /(эм|эм:|э:м:|э:м|хм|х:м|хм:|х:м:|
   э:|а:|а|ам|а:м|ам:|а:м:|м|м:)/ @* *doc*

The query language is sensitive to the character encoding of the cyrillic letters in Russian. The browser-based application ANNIS provides a keyboard tool for different language scripts including Russian cyrillic.

1.2 Syntax of Linear Regression Models

1. FP frequency: model < — lmer(FP frequency $\sim$ language + bilingual + situation + gender + age group + (1 |speaker.id) + (gender |speaker.id) + language:bilingual + language:situation + bilingual:situation + language:gender + language:age group + bilingual:age group + language:bilingual:situation , data)

2. VN ratio: model < — lmer(VN ratio $\sim$ language + bilingual + gender + age group + (1 |speaker.id) + language:bilingual + language:gender + language:age group + bilingual:age group + language:bilingual:age group, data)
